# Physical activity and exercise for chronic pain in adults: an overview of Cochrane Reviews

**DOI:** 10.1002/14651858.CD011279.pub3

**Published:** 2017-04-24

**Authors:** Louise J Geneen, R Andrew Moore, Clare Clarke, Denis Martin, Lesley A Colvin, Blair H Smith

**Affiliations:** OxfordUK; PlymouthUK; Division of Population Health Sciences, University of DundeeNinewells Hospital & Medical SchoolKirsty Semple WayDundeeUKDD2 4DB; Teesside UniversityInstitute of Health and Social CareParksideMiddlesbroughUKTS1 3BA; University of Edinburgh, Western General HospitalAnaesthesia & Pain MedicineEdinburghUK; University of DundeeDivision of Population Health SciencesDundeeUKDD2 4BF

**Keywords:** Adult, Humans, Chronic Pain, Chronic Pain/mortality, Chronic Pain/psychology, Chronic Pain/therapy, Exercise Therapy, Exercise Therapy/adverse effects, Exercise Therapy/methods, Health Services Needs and Demand, Myalgia, Myalgia/etiology, Pain Measurement, Patient Compliance, Quality of Life, Randomized Controlled Trials as Topic, Review Literature as Topic

## Abstract

**Background:**

Chronic pain is defined as pain lasting beyond normal tissue healing time, generally taken to be 12 weeks. It contributes to disability, anxiety, depression, sleep disturbances, poor quality of life, and healthcare costs. Chronic pain has a weighted mean prevalence in adults of 20%.

For many years, the treatment choice for chronic pain included recommendations for rest and inactivity. However, exercise may have specific benefits in reducing the severity of chronic pain, as well as more general benefits associated with improved overall physical and mental health, and physical functioning.

Physical activity and exercise programmes are increasingly being promoted and offered in various healthcare systems, and for a variety of chronic pain conditions. It is therefore important at this stage to establish the efficacy and safety of these programmes, and furthermore to address the critical factors that determine their success or failure.

**Objectives:**

To provide an overview of Cochrane Reviews of adults with chronic pain to determine (1) the effectiveness of different physical activity and exercise interventions in reducing pain severity and its impact on function, quality of life, and healthcare use; and (2) the evidence for any adverse effects or harm associated with physical activity and exercise interventions.

**Methods:**

We searched the*Cochrane Database of Systematic Reviews* (CDSR) on the Cochrane Library (CDSR 2016, Issue 1) for systematic reviews of randomised controlled trials (RCTs), after which we tracked any included reviews for updates, and tracked protocols in case of full review publication until an arbitrary cut‐off date of 21 March 2016 (CDSR 2016, Issue 3). We assessed the methodological quality of the reviews using the AMSTAR tool, and also planned to analyse data for each painful condition based on quality of the evidence.

We extracted data for (1) self‐reported pain severity, (2) physical function (objectively or subjectively measured), (3) psychological function, (4) quality of life, (5) adherence to the prescribed intervention, (6) healthcare use/attendance, (7) adverse events, and (8) death.

Due to the limited data available, we were unable to directly compare and analyse interventions, and have instead reported the evidence qualitatively.

**Main results:**

We included 21 reviews with 381 included studies and 37,143 participants. Of these, 264 studies (19,642 participants) examined exercise versus no exercise/minimal intervention in adults with chronic pain and were used in the qualitative analysis.

Pain conditions included rheumatoid arthritis, osteoarthritis, fibromyalgia, low back pain, intermittent claudication, dysmenorrhoea, mechanical neck disorder, spinal cord injury, postpolio syndrome, and patellofemoral pain. None of the reviews assessed 'chronic pain' or 'chronic widespread pain' as a general term or specific condition. Interventions included aerobic, strength, flexibility, range of motion, and core or balance training programmes, as well as yoga, Pilates, and tai chi.

Reviews were well performed and reported (based on AMSTAR), and included studies had acceptable risk of bias (with inadequate reporting of attrition and reporting biases). However the quality of evidence was low due to participant numbers (most included studies had fewer than 50 participants in total), length of intervention and follow‐up (rarely assessed beyond three to six months). We pooled the results from relevant reviews where appropriate, though results should be interpreted with caution due to the low quality evidence.

**Pain severity:** several reviews noted favourable results from exercise: only three reviews that reported pain severity found no statistically significant changes in usual or mean pain from any intervention. However, results were inconsistent across interventions and follow‐up, as exercise did not consistently bring about a change (positive or negative) in self‐reported pain scores at any single point.

**Physical function:** was the most commonly reported outcome measure. Physical function was significantly improved as a result of the intervention in 14 reviews, though even these statistically significant results had only small‐to‐moderate effect sizes (only one review reported large effect sizes).

**Psychological function and quality of life:** had variable results: results were either favourable to exercise (generally small and moderate effect size, with two reviews reporting significant, large effect sizes for quality of life), or showed no difference between groups. There were no negative effects.

**Adherence to the prescribed intervention:** could not be assessed in any review. However, risk of withdrawal/dropout was slightly higher in the exercising group (82.8/1000 participants versus 81/1000 participants), though the group difference was non‐significant.

**Healthcare use/attendance:** was not reported in any review.

**Adverse events, potential harm, and death:** only 25% of included studies (across 18 reviews) actively reported adverse events. Based on the available evidence, most adverse events were increased soreness or muscle pain, which reportedly subsided after a few weeks of the intervention. Only one review reported death separately to other adverse events: the intervention was protective against death (based on the available evidence), though did not reach statistical significance.

**Authors' conclusions:**

The quality of the evidence examining physical activity and exercise for chronic pain is low. This is largely due to small sample sizes and potentially underpowered studies. A number of studies had adequately long interventions, but planned follow‐up was limited to less than one year in all but six reviews.

There were some favourable effects in reduction in pain severity and improved physical function, though these were mostly of small‐to‐moderate effect, and were not consistent across the reviews. There were variable effects for psychological function and quality of life.

The available evidence suggests physical activity and exercise is an intervention with few adverse events that may improve pain severity and physical function, and consequent quality of life. However, further research is required and should focus on increasing participant numbers, including participants with a broader spectrum of pain severity, and lengthening both the intervention itself, and the follow‐up period.

## Plain language summary

**Physical activity and exercise for chronic pain in adults ‐ an overview of Cochrane Reviews**

**Background**

Chronic (long‐term) pain is pain that has lasted beyond the body's usual healing time. It is often described as pain that has lasted for at least three months. Chronic pain causes many problems, beyond the pain itself, including fatigue, anxiety, depression, and a poor quality of life.

In the past, people with chronic pain were told to rest. However, general advice now is to keep active ‐ whether to affect the pain directly or to combat the other problems associated with it. Therefore, research studies have attempted to examine the effect of physical activity in people with chronic pain.

This overview aimed to bring together and analyse any reviews published by Cochrane that looked at physical activity and exercise studies in any chronic pain condition, including arthritis, back and neck pain, and menstrual (period) pain.

**Key results and quality of the evidence**

In January 2016, we identified 21 Cochrane Reviews which covered 10 different diagnoses (osteoarthritis (a joint disease), rheumatoid arthritis (joint pain and swelling), fibromyalgia (widespread pain condition), low back pain, intermittent claudication (cramping pain in the legs), dysmenorrhoea (period pain), mechanical neck disorders (neck pain), spinal cord injury, postpolio syndrome (a condition occurring in people who have had polio), patellofemoral pain (pain at the front of the knee)). The physical activity or exercise programme used in the trials ranged in frequency, intensity, and type, including land‐ and water‐based activities, those focusing on building strength, endurance, flexibility and range of motion, and muscle activation exercises.

The quality of the evidence was low. This was mostly due to the small numbers of people with chronic pain who participated in each reviewed study. Ideally, a study should have hundreds of people assigned to each group, whereas most of the studies included in the review process here had fewer than 50 people in total.

There was evidence that physical activity reduced the severity of pain, improved physical function, and had a variable effect on both psychological function and quality of life. However, these results were not found in all studies. The inconsistency could be due to the quality of the studies or because of the mix of different types of physical activity tested in the studies. Additionally, participants had predominantly mild‐to‐moderate pain, not moderate‐to‐severe pain.

**Conclusions**

According to the available evidence (only 25% of included studies reported on possible harm or injury from the intervention), physical activity did not cause harm. Muscle soreness that sometimes occurs with starting a new exercise subsided as the participants adapted to the new activities. This is important as it shows physical activity in general is acceptable and unlikely to cause harm in people with chronic pain, many of whom may have previously feared it would increase their pain further.

Future studies should focus on increasing participant numbers, including a wider range of severity of pain (more people with more severe pain), and lengthening both the intervention (exercise programme) itself, and the follow‐up period. This pain is chronic in nature, and so a long‐term intervention, with longer periods of recovery or follow‐up, may be more effective.

## Background

### Description of the condition

Chronic pain has been defined as pain lasting beyond normal tissue healing time, generally taken to be 12 weeks (International Association for the Study of Chronic Pain; Merskey 2011). It contributes to disability, anxiety and depression, sleep disturbances, poor quality of life, and healthcare costs (Leadley 2014; Moore 2014a; Park 2012).

Chronic pain has a weighted mean prevalence in adults of 20% (Breivik 2006; Moore 2014a), which increases as the population ages (32% of adults aged 25 to 34 years, 62% of adults over 75 years; Abdulla 2013; Elliott 1999). This is a greater proportion than people with asthma (To 2012) or diabetes (IDF 2012) in the same population (van Hecke 2013a). The World Health Organization (WHO) recognises chronic pain as a public health problem throughout the world, with one systematic review assessing the growing evidence that the prevalence of chronic pain in the general population is high internationally (34% in low‐income countries and 30% in high‐income countries; Elzahaf 2012). Chronic painful conditions comprise four of the 10 highest ranking conditions for years lived with disability in 2013 (Vos 2015), and are responsible for considerable loss of quality of life and employment, and increased healthcare costs (Moore 2014b). Despite this, the term 'chronic pain' was only added as a MeSH term in MEDLINE in January 2012 (National Library of Medicine), highlighting the relatively small proportion of specific research dedicated to this population.

Certain factors can contribute to an increased risk of chronic pain (female gender, older age, lower socioeconomic status, geographical and cultural background, and genetics; Smith 2007; van Hecke 2013b). Other factors associated with chronic pain conditions are modifiable, such as smoking status, alcohol intake, nutrition, obesity, comorbidities, employment status and occupational factors, and physical activity level (Smith 2007; van Hecke 2013a).

A review of current issues in the treatment of chronic pain strongly suggests that health professionals traditionally focus on biomedical views of pain, utilising pharmacology first and foremost, and sometimes not addressing potential non‐pharmacological approaches such as physical activity and changing attitudes towards chronic pain (Schofield 2011). Guidance often suggests that lifestyle advice is important: for example, the National Institute for Health and Care Excellence (NICE) osteoarthritis guidelines state that "exercise should be a core treatment ... irrespective of age, comorbidity, pain severity and disability. Exercise should include: local muscle strengthening [and] general aerobic fitness" (NICE 2014).

Non‐pharmacological treatments have been developed, investigated, and implemented, with Cochrane Reviews and protocols evaluating the available evidence for psychological, physical, and other non‐medical interventions (e.g. cognitive behavioural and behavioural therapy, Eccleston 2014; Williams 2012; TENS, Nnoaham 2008; low‐impact/intensity movement/exercise therapy, Wieland 2013; dietary, Straube 2015; and patient education, Engers 2008; Gross 2009). While evidence for the effectiveness of these interventions is of variable quantity and quality, the 2013 Scottish Intercollegiate Guideline Network (SIGN) guidelines on the management of chronic pain made strong recommendations on the use of exercise, based on evidence drawn from randomised controlled trials (RCTs), stating: "exercise and exercise therapies, regardless of their form, are recommended in the management of patients with chronic pain" (SIGN 2013).

### Description of the interventions

Physical activity has been defined by the WHO as "any bodily movement produced by skeletal muscles that requires energy expenditure, including activities undertaken while working, playing, carrying out household chores, travelling, and engaging in recreational pursuits" (WHO 2015). WHO also states that "exercise ... is a sub‐category of physical activity that is planned, structured, repetitive, and aims to improve or maintain one or more components of physical fitness" (WHO 2015).

Physical activity for health can take many different forms: it can be structured exercise, such as in classes, gym‐based, or a DVD or programme performed at home; or unstructured and involve adding just a few small activities each day (activities of daily living). Physical activity and exercise can also vary in intensity, duration, and type: aerobic (such as walking) or more focused on increasing flexibility, strength, or balance. Physical activity and exercise can also be taught (or led) by another individual such as an exercise professional, or initiated and maintained through the person's own initiative and motivation.

Both physical activity and exercise can be performed on land or in the water, and can range from whole‐body to localised (body site‐specific) training. Most forms of exercise can also be modified to be performed where there is restricted movement (e.g. in a chair, a bed, or another assistive device).

### How the intervention might work

Physical activity and exercise can be adapted for an individual, and is something people can do to help themselves. It is likely to be associated with minimal adverse effects, such as interactions with medication and potential for abuse in adults with chronic pain, when compared to pharmaceutical and surgical interventions. It is therefore an attractive option to help manage an individual's pain if the systematic reviews show benefit. However, current evidence suggests that simply giving an individual advice to exercise is insufficient to bring about significant change (SIGN 2013), and a badly prescribed intervention that does not consider the individual's conditions and present state of health and fitness, such as one that does not incorporate pacing or gradual progression, may bring about adverse events such as pain 'flare‐ups', or lead to cardiac or respiratory events (American College of Sports Medicine 2007). This suggests that supervised or structured interventions may be more fruitful, though this is currently unconfirmed.

Since the 1980s, primary care physician advice for treating pain has changed, moving away from "rest", to minimising or eliminating bedrest and instead remaining active (back pain, Waddell 1987). Exercise may have specific benefits in reducing the severity of chronic pain, as well as more general benefits associated with improved overall physical and mental health, and physical functioning of people with chronic pain, as depression (Finan 2013), deconditioning (Bousema 2007), and obesity are commonly observed in these people (headache/migraine, Bigal 2012; fibromyalgia, Ursini 2011). For example, studies have revealed that a single bout of exercise increases the production of endogenous opioids, leading to transient anti‐nociception in both animals and humans, and repeated exercise produces long‐lasting anti‐nociception in otherwise untreated animals (Stagg 2011). Aerobic exercise is also strongly linked to weight loss (Messier 2013), which in turn has implications for the management of chronic pain as the pressure on joints is reduced. Alternatively, resistance exercise, or other forms of strength training, can improve the person's capacity to support bone and cartilage through improved musculature supporting movement around a joint, with potential to relieve stiffness (Mayer 2008) and bringing about some pain relief. Resistance training through repetitive full range‐of‐motion exercise around the lumbar spine (in chronic low back pain) may affect disc metabolism itself, with the possibility that the exercise programme could improve metabolic exchange in the lumbar discs and aid in repair (Mooney 2006). Training to improve balance and flexibility also has benefits as it reduces the risk of falls, and the potential for further pain or injury (Harvard 2013).

### Why it is important to do this overview

If physical activity and exercise interventions are shown to effectively and safely reduce pain intensity or frequency (or both), they are likely to be a preferable alternative or adjunct therapy to pharmacological/surgical treatments for chronic pain. The interventions could promote personal involvement of individuals in the management of their pain, thus increasing self‐efficacy and the ability to self‐manage. In turn this could lead to an increase in overall quality of life and a consequent reduction in healthcare use. In addition, exercise is of great importance for cardiovascular (Vigorito 2014) and bone health (Sakuma 2012). Reduced physical function and consequent lack of mobility in people with chronic pain is associated with increased all‐cause and cardiovascular mortality (Nüesch 2011), with other studies linking severe chronic pain to general increased all‐cause mortality (Moore 2014a; Torrance 2010).

Physical activity and exercise programmes are increasingly being promoted and offered in various healthcare systems (American College of Sports Medicine (ACSM) 'Exercise is Medicine' global pledge at the Inaugural World Congress 2010) and for a variety of chronic pain conditions, including arthritis (Fransen 2014; Silva 2010), fibromyalgia (Busch 2013), and dysmenorrhoea (Brown 2010). At this stage it is important to establish the efficacy and safety of these programmes, and furthermore to address the critical factors that determine their success or failure.

It is therefore important to identify whether (and how) exercise interventions can be effectively and safely applied in people with chronic pain.

With a number of systematic reviews published by Cochrane evaluating the effectiveness of exercise in various painful conditions, it is timely and important to bring together all relevant published information to evaluate the current evidence, and identify the availability and quality of evidence‐based exercise interventions. This overview will determine the extent to which the published systematic reviews have accurately assessed the evidence for exercise in chronic pain conditions/syndromes, which will help to direct future guidelines and identify current research gaps.

## Objectives

To provide an overview of Cochrane Reviews of adults with chronic pain to determine (1) the effectiveness of different physical activity and exercise interventions in reducing pain severity and its impact on function, quality of life, and healthcare use; and (2) the evidence for any adverse effects or harm associated with physical activity and exercise interventions.

## Methods

### Criteria for considering reviews for inclusion

We included only systematic reviews of RCTs of physical activity and exercise in participants with chronic pain, and published in the *Cochrane Database of Systematic Reviews*. The included reviews had to fulfil the following criteria:

#### Participants

Adults (aged 18 years and over) reporting chronic non‐cancer pain, including persistent (e.g. chronic back pain, fibromyalgia) and intermittent (e.g. migraine, dysmenorrhoea) pain, for at least three months (12 weeks) in any body site.

#### Intervention

Reviews of RCTs assessing physical activity or exercise as the intervention (any reviews where that assessed physical activity or exercise as a stand‐alone intervention). This included physical activity interventions that could be initially taught by an exercise professional, or involve periodical/ongoing supervision.

#### Exclusions

Interventions not deemed physical activity or exercise using the WHO definition, such as manipulation, mobilisation, or passive movement. Any multi‐modal interventions were excluded if physical activity/exercise could not be assessed for effect (the effect of exercise must have been measured distinctly).

#### Comparison

Usual care, waiting list control, placebo/sham treatment, other treatment, or a combination of treatments (as long as the effect of exercise could be measured distinctly).

#### Primary outcome

- self‐reported pain (severity).

This could be presented and analysed as change on a continuous scale, the proportion of participants who 'responded', or, ideally, in a dichotomised format as the proportion of participants in each group who achieved a predetermined threshold of improvement (e.g. outcome in individual participants of at least 50% pain intensity reduction, or no worse than mild pain, at the end of the trial, with at least 30% pain intensity reduction as a secondary outcome, or recovery; Moore 2013).

#### Secondary outcomes

- Physical function (objectively or subjectively measured).
- Psychological function.
- Quality of life.
- Adherence to the prescribed intervention.
- Healthcare use/attendance.
- Adverse events (not death).
- Death.

Reviews may not always report specifically on activity or exercise for chronic pain in adults. We anticipated two possible circumstances which might have arisen.

- A review included some interventions of interest or reported only some outcomes of interest. In this case we extracted the interventions and outcomes of interest, but we did not include interventions or outcomes outside the scope of this overview.
- Reviews occasionally included papers that included children and adults together, but the results for adults were not reported or analysed separately in the included papers or the review. In this case we made a judgement as to whether the review could be included based on the proportion of adults. Our intention was to include only those reviews where more than 80% of participants were adults.

### Search methods for identification of reviews

We searched the *Cochrane Database of Systematic Reviews* (CDSR), 2016, Issue 1, on the Cochrane Library for relevant reviews using the search strategy: (*pain or migraine or headache*) and (*exercise or activity or physical*)*.* We did not seek non‐Cochrane reviews.

### Data collection and analysis

Two overview authors (LG, CC) independently carried out searches and selected reviews for inclusion. Disagreements were resolved through discussion, and a third overview author (RAM) acted as arbitrator where necessary.

Two overview authors (independently carried out assessment of methodological quality (LG, CC), and extracted data (LG, RAM). Any disagreements were resolved through discussion, or involving a third overview author if necessary (DM).

One overview author (LG) tracked results of the search for the most up to date version of each review and protocol that fulfilled the inclusion criteria.

#### Selection of reviews

Included reviews assessed RCTs of the effects of exercise for pain management in adults (as defined by individual reviews), compared with any of the listed comparators, and included:

- a clearly defined clinical question;
- details of inclusion and exclusion criteria;
- details of databases searched and relevant search strategies;
- participant‐reported pain severity (primary outcome measure);
- summary results for at least one other desired outcome.

#### Data extraction and management

Two overview authors (LG, RAM) independently extracted data from the included review using a standardised data extraction form and checked for agreement prior to entry into Microsoft Excel for Windows. We did not extract data from reports included in the reviews again, neither did we undertake any re‐analysis of data from reviews. Data were not entered for analysis into Cochrane's statistical software due to the lack of relevant and comparable data (RevMan 2014).

We collected the following information (where available) from the reviews:

- number of included studies and participants;
- intervention (exercise or activity type) and dose (frequency/intensity);
- comparator;
- condition treated;
- time of assessment;
- duration of follow‐up;
- relevant outcomes.

Where possible we extracted risk ratio (RR), number needed to treat for an additional beneficial outcome (NNTB), mean difference (MD), and standardised mean difference (SMD), and other relevant statistical data for the primary and secondary outcomes. This included:

- obtaining 50% pain relief (participant‐reported);
- obtaining any other measure of 'improvement' (participant‐reported);
- adverse events;
- death;
- withdrawals.

#### Assessment of methodological quality of included reviews

##### Quality of included reviews

Two overview authors (LG, CC) independently assessed each included review to see if it satisfied the criteria specified in the 'assessment of multiple systematic reviews' (AMSTAR) measurement tool (Shea 2007), for rigorous methodological quality. Arbitration by a third overview author (DM) was necessary for some fields.

High quality reviews were required to fulfil each of the established AMSTAR criteria (further criteria to fulfil each field is listed in Table 1).

**1:** AMSTAR tool to assess the methodological quality of systematic reviews

**Criteria**	**Specific requirements (possible answers: yes, no, cannot answer, not applicable)**
1. Was an 'a priori' design used?	The research question and inclusion criteria should be established before the conduct of the review. *Note: need to refer to a protocol, ethics approval, or predetermined/a priori published research objectives to score a* "*yes.*"
2. Was there duplicate study selection and data extraction?	There should be at least 2 independent data extractors and a consensus procedure for disagreements should be in place. *Note: 2 people do study selection, 2 people do data extraction, consensus process or 1 person checks the other person's work.*
3. Was a comprehensive literature search performed?	At least 2 electronic sources should be searched. The report must include years and databases used (e.g. CENTRAL, MEDLINE, and Embase). Keywords or MeSH terms (or both) must be stated and where feasible the search strategy should be provided. All searches should be supplemented by consulting current contents, reviews, textbooks, specialised registers, or experts in the particular field of study, and by reviewing the references in the studies found. *Note: if at least 2 sources + 1 supplementary strategy used, select* "*yes*"*(Cochrane register/ CENTRAL counts as 2 sources; a grey literature search counts as supplementary).*
4. Was the status of the publication (i.e. grey literature) used as inclusion criteria?	The authors should state that they searched for reports regardless of their publication type. The authors should state whether or not they excluded any reports (from the systematic review), based on their publication status, language, etc. *Note: if review indicates that there was a search for* "*grey literature*"*or* "*unpublished literature,*"*indicate* "*yes.*"*SIGLE database, dissertations, conference proceedings, and trial registries are all considered grey for this purpose. If searching a source that contains both grey and non‐grey, must specify that they were searching for grey/unpublished literature.*
5. Was a list of studies (included and excluded) provided?	A list of included and excluded studies should be provided. *Note: acceptable if the excluded studies were referenced. If there was an electronic link to the list but the link is no longer active, select* "*no.*"
6. Were the characteristics of the included studies provided?	In an aggregated form such as a table, data from the original studies should be provided on the participants, interventions, and outcomes. The ranges of characteristics in all the studies analysed, e.g. age, race, sex, relevant socioeconomic data, disease status, duration, severity, or other diseases should be reported. *Note: acceptable if not in table format as long as they are described as above.*
7. Was the scientific quality of the included studies assessed and documented?	'A priori' methods of assessment should be provided (e.g. for effectiveness studies if the author(s) chose to include only randomised, double‐blind, placebo‐controlled studies, or allocation concealment as inclusion criteria); for other types of studies alternative items will be relevant. *Note: can include use of a quality scoring tool or checklist, e.g. Jadad scale, risk of bias, sensitivity analysis, etc., or a description of quality items, with some type of result for EACH study (*"*low*"*or* "*high*"*is acceptable, as long as it is clear which studies scored* "*low*"*and which scored* "*high;*"*a summary score/range for all studies is not acceptable).*
8. Was the scientific quality of the included studies used appropriately in formulating conclusions?	The results of the methodological rigor and scientific quality should be considered in the analysis and the conclusions of the review, and explicitly stated in formulating recommendations. *Note: might say something such as* "*the results should be interpreted with caution due to poor quality of included studies.*"*Cannot score* "*yes*"*for this question if scored* "*no*"*for question 7.*
9. Were the methods used to combine findings of studies appropriate?	For the pooled results, a test should be done to ensure the studies were combinable, to assess their homogeneity (i.e. Chi^2^ test for homogeneity, I^2^ statistic). If heterogeneity exists, a random‐effects model should be used or the clinical appropriateness of combining should be taken into consideration (i.e. is it sensible to combine?), or both. *Note: indicate* "*yes*"*if they mention or describe heterogeneity, i.e. if they explain that they cannot pool because of heterogeneity/variability between interventions.*
10. Was the likelihood of publication bias assessed?	An assessment of publication bias should include a combination of graphical aids (e.g. funnel plot, other available tests) or statistical tests (e.g. Egger regression test), or both. *Note: if no test values or funnel plot included, score* "*no.*"*Score* "*yes*"*if they mention that publication bias could not be assessed because there were fewer than 10 included studies.*
11. Was the conflict of interest stated?	Potential sources of support should be clearly acknowledged in both the systematic review and the included studies. *Note: to get a* "*yes,*"*must indicate source of funding or support for the systematic review AND for each of the included studies.*

For each review we also planned to assess the likelihood of publication bias by calculating the number of participants in studies with zero effect (relative benefit of one) that would be needed to give an NNTB too high to be clinically relevant (Moore 2008). In this case we would have considered an NNTB of 10 or greater for the outcome of participant‐reported pain relief of 30% or greater to be the cut‐off for clinical relevance. This method is used as statistical tests for the presence of publication bias have been shown to be unhelpful (Thornton 2000). However, assessment of publication bias was not possible due to the lack of specificity of the populations included within the reviews, and so we were unable to extract comparable data.

##### Quality of evidence in included reviews

We planned to use two main indicators for the quality of evidence: all included reviews must have used only primary studies that were both randomised and double‐blind, so minimising the risk of bias from these items; and all included reviews must have included only people with at least moderate pain intensity at baseline (visual analogue scale greater than 30/100, categorical rating scale greater than 1/3, and numerical rating scale greater than 3/10, Collins 1997), providing a sensitive assay of intervention efficacy.

Subsequently, we planned to analyse data for each painful condition in three tiers, according to outcome and freedom from known sources of bias.

- The first tier used data meeting current best standards, where studies reported the outcome of at least 50% pain intensity reduction from baseline (where 50% was the cut‐off for a dichotomous (yes/no) outcome: was a 50% reduction in pain observed?), or its equivalent, without using last observation carried forward (LOCF) or other imputation method for dropouts, reported an intention‐to‐treat (ITT) analysis, lasted eight or more weeks, had a parallel‐group design, and had at least 200 participants (preferably at least 400) in the comparison (Moore 2010). These top‐tier results were usually reported first.
- The second tier used any available data, but where one or more of these conditions were not met, for example reporting at least 30% pain intensity reduction, using LOCF or a completer analysis, lasting four to eight weeks, and where the numbers of participants were at least 200.
- A third tier of evidence related to small amounts of data (fewer than 200 participants), or short studies of less than four weeks, or where there was obvious major heterogeneity between studies, or where there were other shortcomings in allocation concealment, considerable attrition, and incomplete outcome data. For this third tier of evidence, no data synthesis was reasonable, and may have been misleading, but an indication of beneficial effects might be possible.

This overview examined the quality of all included reviews according to current best standards for reporting in pain. These included the attempt and ability of the reviews to identify studies/interventions with the maximum evidence of effectiveness, and minimum risk of bias, including the reporting of the following.

- Outcomes in trials of the proportion of participants obtaining at least 50% pain intensity reduction, or no worse than mild pain, at the end of the trial (with at least 30% pain intensity reduction as a secondary outcome). We did not consider the use of mean changes in pain scores as high quality because responses to pain interventions are not Gaussian, and few people have the mean response.
- Duration of included studies of eight weeks or longer.
- Imputation method of baseline observation carried forward (BOCF), LOCF, or worst observation carried forward (WOCF) if adverse event withdrawals were similar in active and control groups.
- At least 200 participants per treatment group in included studies, with at least two trials, as a minimum criterion for trustworthiness of any analysis. Pooled analysis of small studies may be considered good quality if at least 400 participants were involved, but we regarded these as being potentially subject to bias.

We extracted the 'Risk of bias' as assessed by the original review authors from included reviews. Counts of low risk of bias were extracted from relevant studies in the included reviews and tabulated under the following headings to evaluate the proportion of studies achieving a low risk of bias for each:

- random sequence generation (selection bias);
- allocation concealment (selection bias);
- blinding of participants and personnel (performance bias);
- blinding of outcome assessment (detection bias);
- incomplete outcome data (attrition bias);
- selective reporting (reporting bias);
- sample size;
- any other biases.

#### Data synthesis

Additional quantitative analyses were not required, since we only considered results from properly conducted (Cochrane) reviews. The aim was to concentrate on specific outcomes such as the proportion of participants with at least 50% pain relief, all‐cause or adverse event discontinuations, or serious adverse events, and to explore how these can be compared across different treatments for the same condition. We planned to compare only like with like (where possible); for example in study duration, which can be an additional source of bias if insufficient in length (Moore 2010).

However due to the limited data available, we were unable to directly compare and analyse interventions, and have instead reported the evidence qualitatively only. We had also planned to employ subgroup analyses assessing age, condition, and intervention type/intensity, though this was not feasible using the available data from included reviews. For this reason we have also been unable to include a 'Summary of findings' table as planned and stated in the protocol.

Importantly, we have tried to highlight issues of low trial quality, inadequate size, and whether trials were truly valid for the particular condition in making between‐therapy comparisons.

We approached each review with four main questions/focus, and extracted data accordingly.

- Did they report exercise versus non‐exercise studies?
- Did the review or studies included in the review (or both) have low risk of bias?
- Did they have our main outcome?
- What were the actual intervention/s included in the review?

## Results

We included 21 reviews with 381 included studies, totalling 37,143 participants. Of these, 264 studies (19,642 participants) examined exercise versus no exercise/minimal intervention in adults with chronic pain (the focus of this overview) and so were used in the qualitative analysis.

### Description of included reviews

The search strategy was performed in the Cochrane Library only, and revealed 475 potentially relevant titles, of which 75 were assessed as full papers.

The search was undertaken on 31 January 2016 (CDSR 2016, Issue 1), after which any included reviews were tracked for updates, and protocols were followed in case of full review publication until 21 March 2016 (CDSR 2016, Issue 3).

All extracted data and methodological quality assessment were taken from the most recent published version of the full review.

Ultimately, of the 75 titles requiring further assessment, 10 were reviews at protocol stage only (five of which have potential to be included once published as a full review, one which was unclear, and four that were excluded based on information within the protocol). Hence, we excluded 54 titles (10 protocols and 44 full reviews; Figure 1), reasons for which are listed in Table 2.

**1:**
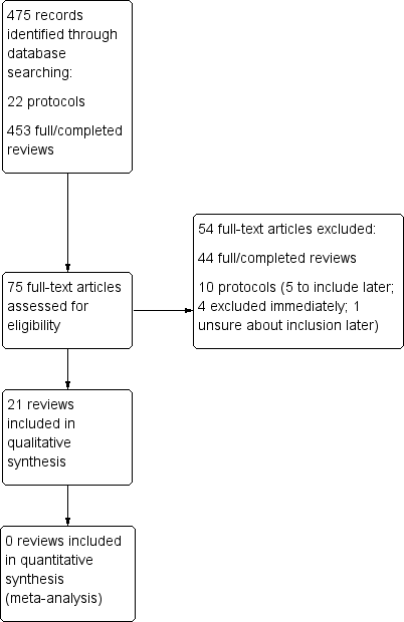
Study flow diagram.

**2:** Reasons for exclusion

**Review**	**Reason for exclusion from overview**
Aggarwal 2011	Not exercise/physical activity
Brønfort 2015	Protocol stage only ‐ possibly include when published as full review
Bierma‐Zeinstra 2011	Protocol stage only ‐ exclude when published as full review
Brønfort 2014	Withdrawn from the Cochrane Library
Choi 2010	Not chronic using definition of > 3 months
Craane 2006	Protocol stage only ‐ possibly include when published as full review
Dagfinrud 2008	Physiotherapy ‐ required therapist to perform intervention
Dahm 2010	Acute pain, not chronic. Intervention was advice
Dal Bello‐Haas 2013	Malignant condition
de Souza 2012	Drug‐ and surgery‐based interventions
Fokkenrood 2013	Did not include RCTs (excluded studies with control groups)
Franke 2015	Not exercise/physical activity
Green 2003	Physiotherapy ‐ required therapist to perform intervention
Gross 1998	Withdrawn from the Cochrane Library
Gross 2012	Not exercise/physical activity
Gross 2015b	Not exercise/physical activity
Hayden 2012	Protocol stage only ‐ possibly include when published as full review
Heintjes 2003	Withdrawn from the Cochrane Library January 2015
Henschke 2010	Not exercise/physical activity
Heymans 2004	Exercise could not be assessed as stand‐alone intervention
Hilde 2006	Withdrawn from the Cochrane Library
Hoving 2014	No exercise intervention, and no pain outcome measure
Hurley 2013	Protocol stage only ‐ exclude when published as full review
IJzelenberg 2011	Protocol stage only ‐ exclude when published as full review
Jones 2000	Drug‐based interventions
Jordan 2010	Intervention to improve adherence to exercise, not exercise itself
Kamper 2014	Exercise could not be assessed as stand‐alone intervention
Karjalainen 1999	Exercise could not be assessed as stand‐alone intervention
Karjalainen 2003	Exercise could not be assessed as stand‐alone intervention
Larun 2016	Chronic fatigue, not chronic pain
Liddle 2015	Pain in pregnancy only, not chronic pain
Liu 2013	Protocol stage only ‐ unsure about inclusion when published as full review
Miller 2014	Protocol stage only ‐ exclude when published as full review
Moi 2013	Exercise could not be assessed as stand‐alone intervention
O'Brien 2004	No pain outcome measure
O'Connell 2013	Overview of reviews, not systematic review
Østerås 2013	Protocol stage only ‐ possibly include when published as full review
Page 2012	No pain outcome measure
Page 2014	Manual therapy ‐ required therapist to perform intervention
Peters 2013	Exercise could not be assessed as stand‐alone intervention
Preston 2004	No pain outcome measure
Proctor 2007	Exercise could not be assessed as stand‐alone intervention
Radner 2012	Drug‐based interventions
Regnaux 2014	Protocol stage only ‐ possibly include when published as full review
Richards 2012	Not exercise/physical activity
Riemsma 2003	Not exercise/physical activity
Schaafsma 2013	No pain outcome measure
Steultjens 2004	Occupational therapy ‐ exercise could not be assessed as stand‐alone intervention
Stones 2005	Exercise cannot be assessed as stand‐alone intervention
Takken 2008	Aged < 18 years ‐ not adults
van Dessel 2014	Not chronic pain and no specific pain outcome measure
White 2004	No pain outcome measure
Williams 2012	Not exercise/physical activity
Zammit 2010	Surgery or required therapist to perform intervention

RCT: randomised controlled trial.

Detailed information about the included reviews is available in Table 3. Trial and participant number, age, and gender distribution is reported in Table 4.

**3:** Characteristics of included reviews

**Review and Cochrane Review Group**	**Assessed as up to date**	**Chronic pain condition**	**Duration of pain/ diagnosis**	**Intervention description**	**Control description**	**Outcomes with data reported**	**Time points reported**
Bartels 2007 Cochrane Musculoskeletal Group	Aug 2007	Hip or knee OA	Not reported	All types of exercises developed in the therapeutic/heated indoor pool (ROM, dynamics, aerobics, etc.) were permitted.	No treatment or other treatment.	Function, quality of life, mental health, pain, adverse events	Post‐intervention (immediate), 6‐month follow‐up
Bidonde 2014 Cochrane Musculoskeletal Group	Oct 2013	Fibromyalgia	12 yr (range 6 to 24)	Aquatic exercise training intervention defined as "exercise conducted in a vertical standing position."	Treatment as usual, physical activity as usual, wait list control, placebo or sham, education‐only, water immersion‐only, and attention only.	Multi‐dimensional function (wellness), self‐reported physical function (wellness), pain (symptoms), stiffness (symptoms), muscle strength (physical fitness), submaximal cardiorespiratory function (physical fitness), withdrawals (safety and acceptability), adverse effects (safety and acceptability)	Post‐intervention (4 to 32 wk)
Boldt 2014 Cochrane Injuries Group	Mar 2011	Spinal cord injury	Mean 66 months, and 1 to 24 yr when reported	"Exercise": stretching and strengthening exercises aimed at mobilising painful shoulder joint.	Wait list control or no intervention.	Pain, depression, quality of life, adverse effects	Short term (within 24 hours of last intervention, i.e. post‐intervention) and intermediate term (1 to 6 wk post‐intervention) and long term (> 6 wk post‐intervention)
Brown 2010 Cochrane Menstrual Disorders and Subfertility Group	Aug 2009	Primary dysmenorrhoea in the majority (≥ 50%) of cycles	Ongoing/not appropriate	12‐wk walk or jog training programme at an intensity of 70% to 85% of the HR range. Training for 3 days/wk and duration of aerobic phase was 30 minutes with 15‐minute warm‐up and cool‐down periods.	Asked not to exercise during the experimental period.	Pain: menstrual disorders questionnaire (MDQ) score	Ongoing ‐ over 3 menstrual cycles
Busch 2007 Cochrane Musculoskeletal Group	Aug 2007	Fibromyalgia	Not reported	Exercise‐only interventions included aerobic‐only training, strength‐only training, flexibility‐only training, or mixed exercise‐only interventions.	"Untreated."	Pain, global wellbeing, objectively measured physical function	Post‐intervention (strength exercise 21 wk, aerobic exercise 6 to 23 wk)
Busch 2013 Cochrane Musculoskeletal Group	Mar 2013	Fibromyalgia	mean range from 4 yrs (SD 3.1) to 12 yrs (SD 4)	Defined resistance training as exercise performed against a progressive resistance on a minimum of 2 days/wk (on non‐consecutive days) with the intention of improving muscle strength, muscle endurance, muscle power, or a combination of these.	Untreated control conditions (treatment as usual, activity as usual, wait list control, and placebo), other types of exercise or physical activity interventions (e.g. aerobic, flexibility), and other resistance training interventions (head‐to‐head comparisons).	Multi‐dimensional function, self‐reported physical function, pain, tenderness, muscle strength, adverse effects, all‐cause attrition	Post‐intervention, follow‐up (12 wk) in 1 study only
Cramp 2013 Cochrane Musculoskeletal Group	Oct 2012	Rheumatoid arthritis	Not reported	Included pool‐based therapy (twice/wk, moderate intensity, music‐paced), yoga (6 wk, twice/wk, 1.5‐hour sessions), dynamic strength training (home‐based after inpatient programme, all main muscle groups using dumbbells and elastic bands), stationary cycling (70% HRmax, 5 minute excluding: 1‐minute of rest, increased duration), low‐impact aerobics (class at fitness centre and video at home, individual HR targets), tai chi (1‐hour group sessions).	"Could have been placebo, an alternative intervention (pharmacological or non‐pharmacological) or usual care."	Fatigue, pain, anxiety, depression, disability, tender and swollen joints, adverse events	Post‐intervention (only a single time point analysed)
Fransen 2014 Cochrane Musculoskeletal Group	May 2013	Hip OA	Not reported	Any land‐based therapeutic exercise regimens aiming to relieve the symptoms of hip OA, regardless of content, duration, frequency, or intensity. This included any exercise designed to improve muscle strength, range of joint movement or aerobic capacity (or combinations of the three). Programmes could be designed and supervised by physiotherapists or other professionals, or provided as a home programme with minimal monitoring.	Wait‐list control, usual care, GP education.	Self‐reported pain, physical function, quality of life, withdrawal or dropouts, adverse events	post‐intervention (immediate in 9/10 studies) follow‐up 3 to 6 months
Fransen 2015 Cochrane Musculoskeletal Group	May 2013	Knee OA	Often not reported: some less than 1yr, others over 10yr	"land‐based therapeutic exercise." Along with delivery mode and content, treatment 'dosage' (duration, frequency, intensity) varied widely between studies.	No exercise: active (any no‐exercise intervention) or no treatment (including waiting list).	Knee pain, self‐reported physical function, quality of life	Immediately at the end of treatment (post‐treatment), 2 to 6 months after cessation of monitored study treatment and longer than six months after cessation of monitored study treatment
Gross 2015a Cochrane Back Group	May 2014	Mechanical neck disorders	"Chronic" (not subacute or acute)	Cervical stretch/ROM exercises + cervical/scapulothoracic strengthening + static/dynamic cervical/shoulder stabilisation.	Wait list control.	Pain intensity, function, quality of life, global perceived effect, adverse effects	Immediately post‐treatment (≤ 1 day), short‐term follow‐up (1 day to 3 months), intermediate‐term follow‐up (3 months up to, but not including, 1 yr), and long‐term follow‐up (≥ 1 yr)
Han 2004 Cochrane Musculoskeletal Group	Apr 2004	Rheumatoid arthritis	Not reported	Only trials of exercise programmes with tai chi instruction or incorporating principles of tai chi philosophy.	Not reported.	Function, tender and swollen joints, ROM, strength, enjoyment, withdrawals, adverse effects	Post‐intervention (8 to 10 wk)
Hayden 2005 Cochrane Back Group	Sep 2004	Non‐specific low back pain	Chronic, i.e. longer than 12 wk: 5.6 yr (95% CI 3.4 to 7.8)	Exercise therapy defined as "a series of specific movements with the aim of training or developing the body by a routine practice or as physical training to promote good physical health;" only 54% adequately described the exercise intervention.	No exercise: no treatment or placebo treatment, other conservative therapy, or another exercise group.	Pain, functional ability, work status, global assessment, adverse events	Earliest, 6 wk, 6 months, 12 months
Hurkmans 2009 Cochrane Musculoskeletal Group	Jun 2009	Rheumatoid arthritis	5 to 14 yr	Dynamic exercise programmes ‐ aerobic capacity and muscle strength training; short‐term muscle strength training (high quality); short‐term dynamic exercise to improve aerobic capacity (not high methodological quality); exercise frequency of at least 20 minutes twice a week. Duration of exercise programme at least 6 wk (duration < 3 months was considered short‐term; duration > 3 months was considered long‐term). Exercise programme performed under supervision. Aerobic exercise intensity at least 55% of the maximum HR; or intensity starting at 40% to 50% of the maximum oxygen uptake reserve or HR maximum reserve. Furthermore, the intensity was increased up to 85% during the intervention. Progressively strengthening exercise loads starting at 30% to 50% and increasing to 80% of maximum (defined as the percentage of either 1 repetition maximum, 1 MVC, maximum speed, or as maximal subjective exertion).	Not reported	Functional ability, aerobic capacity, muscle strength, safety (pain and radiological damage)	Follow‐up (12 wk and 24 months)
Koopman 2015 Cochrane Neuromuscular Group	Jul 2014	Postpolio syndrome (PPS)	Not reported	Exercise therapy (e.g. aerobic exercise, muscle strengthening exercise, respiratory muscle training, warm climate training, hydro training).	Placebo, usual care or no treatment.	Self‐perceived activity limitations, muscle strength, muscle endurance, fatigue, pain, adverse events (minor and serious)	3 and 6 months
Lane 2014 Cochrane Peripheral Vascular Diseases Group	Sep‐2013	intermittent claudication	not reported	Any exercise programme used in the treatment of intermittent claudication was included, such as walking, skipping and running. Inclusion of trials was not affected by the duration, frequency or intensity of the exercise programme but these issues were taken into account in the meta‐analysis	Exercise was compared to six different modes of treatment, the most common being usual care or placebo. Two early trials compared exercise with placebo tablets but in more recent studies usual care was used as the control comparator. Exercise was compared with the following drug therapies: antiplatelet agents pentoxifylline, iloprost, and vitamin E. One study compared exercise with pneumatic foot and calf compression.	maximal walking time, pain‐free walking time, pain‐free walking distance, maximum walking distance, ankle brachial index (ABI), peak exercise calf blood flow, mortality, amputation	Post‐intervention, 3‐month follow up, six‐month follow up
Lauret 2014 Cochrane Peripheral Vascular Diseases Group	Jul 2013	Intermittent claudication	Not reported	Supervised walking programme needed to be supervised at least twice a week for a consecutive 6 wk of training.	Alternative exercise.	Maximum walking distance (METs), pain‐free walking distance (METs), health‐related quality of life and functional impairment	n/a
Regnaux 2015 Cochrane Musculoskeletal Group	Jun 2014	Hip or knee OA	> 6 months	High‐intensity physical activity or exercise programme.	Low‐intensity physical activity or exercise programme and control (no‐exercise) group in 1 study.	Pain, physical function, quality of life, adverse effects (related to intervention), severe adverse events or withdrawal (due to intervention)	Post‐intervention, intermediate term (6 to 12 months), long‐term (over 12 months) follow‐up
Saragiotto 2016 Cochrane Back and Neck Group	Apr 2015	Low back pain	> 12 wk	MCE: activation of the deep trunk muscles, targeting the restoration of control and co‐ordination of these muscles.	Placebo, no treatment, another active treatment, or when MCE was added as a supplement to other interventions. When MCE was used in addition to other treatments, it had to represent at least 50% of the total treatment programme to be included.	Pain intensity and disability, function, quality of life, global impression of recovery, return to work, adverse events and recurrence	Post‐intervention, short term (4 to 10 wk), intermediate term (3 to 6 months), long term (12 to 36 months)
Silva 2010 Cochrane Musculoskeletal Group	Jun 2009	Rheumatoid arthritis	No studies found	Balance training (proprioceptive training).	No intervention or other intervention.	ACR‐50, pain, disease activity score (DAS), Health Assessment Questionnaire (HAQ for function), gait, adverse effects, discontinuation rate	n/a
van der Heijden 2015 Cochrane Bone, Joint and Muscle Trauma Group	May 2014	Adolescents and adults with patellofemoral pain	3 wk to 8 months (as minimum requirement); reported pain 4 wk to 9 yr	Exercise therapy for patellofemoral pain syndrome; exercises could be performed at home or under supervision of a therapist ‐ various descriptions in the included trials, including knee exercises, hip and knee exercises, home exercises, supervised exercises, closed kinetic chain, open kinetic chain.	No treatment, placebo, or waiting list controls. This also included 'exercise therapy + another intervention (e.g. taping) versus the other intervention alone (e.g. taping).'	Pain during activity, usual pain, functional ability, recovery	4‐ to 12‐wk follow‐up (short term) and 16 wk to 12 months (long term)
Yamato 2015 Cochrane Back Group	Mar 2014	Low back pain	Acute, subacute, chronic (i.e. no minimum)	Explicitly stated as based on Pilates principles, or the therapists who provided the interventions had previous training in Pilates exercises or the therapists were described as certified Pilates instructors	No intervention, placebo, or other interventions.	Pain intensity, disability, global impression of recovery, quality of life, return to work, adverse effects	Short term (4 to 8 wk), intermediate term (3 to 6 months)

ACR: American College of Rheumatology; GP: general practitioner; HR: heart rate; MCE: motor control exercise; MET: metabolic equivalents; n/a: not applicable; OA: osteoarthritis; ROM: range of motion; wk: week; yr: year.

**4:** Further characteristics of included reviews

**Review**	**Number of trials included**	**Total number of participants**	**Gender distribution**	**Participants ages**
Bartels 2007	6 (4 exercise vs no exercise)	800 (674 exercise vs no exercise)	50% to 86% Female	Means ranged from 66 to 71 yr
Bidonde 2014	16 (9 exercise vs no exercise)	881 (519 exercise vs no exercise)	513 female, 6 male	Means ranged from 46.3 to 48.3 yr
Boldt 2014	16 (3 exercise vs no exercise)	616 (149 exercise vs no exercise)	115 male, 41 female across 3 studies	Range 19 to 65 yr and mean 35 to 45 yr
Brown 2010	1	36	100% female	Not reported
Busch 2007	34 (in meta‐analysis ‐ strength training vs control: 2; aerobic training vs control: 4)	2276 total (in meta‐analysis ‐ strength: 47, aerobic: 269)	96.4% female when reported (in 2197 participants)	Range reported as 27.5 to 60.2 yr
Busch 2013	5 studies as 7 publications (exercise vs control: 3 publications, 2 studies)	219 with fibromyalgia (exercise vs control: 81)	100% female	Not reported
Cramp 2013	24 (only 6 using physical activity interventions)	2882 (physical activity interventions: 371)	"A higher percentage of females"… when reported	"Mainly within the fifth decade"
Fransen 2014	10	> 549	75% to 80% female when reported	58 to 70 yr (means) when reported
Fransen 2015	54	5362	When reported 55% to 100% female	When reported mean age 60 to 70 yr
Gross 2015a	27 (16 chronic pain)	2485	Not reported	Not reported
Han 2004	4 (3 RCTs). Pain not reported in any included study	206 total; pain not reported in any included study	Not reported	Range 38 to 72 yr
Hayden 2005	61 (43 chronic low back pain)	6390 (3907 chronic low back pain)	Chronic: 46% male (95% CI 39 to 52)	Chronic: 42 yr (95% CI 40 to 44)
Hurkmans 2009	8 RCTs (5 exercise vs no‐exercise)	575	"Mainly female"	52 yr
Koopman 2015	13 (2 exercise vs no exercise)	675 (68 exercise vs no exercise) ‐ 1 study used 3 arms (no treatment in cold, exercise in cold, exercise in warm; we have excluded the warm exercise arm as cannot compare directly to the control)	˜ 25% male	Mean 58 and 65 yr
Lane 2014	30	1822 total	Not reported	Mean > 65 yr
Lauret 2014	5 (0 for exercise vs no exercise)	184 (0 for exercise vs no exercise)	n/a	n/a
Regnaux 2015	6 (1 for exercise vs no exercise) only 1 study that had a no exercise control	656 (102 for exercise vs no exercise)	79 female	62.6 yr
Saragiotto 2016	29 (7 for exercise vs no exercise/minimal intervention)	2431 (671 for exercise vs no exercise)	"Mixed"	Median 40.9 yr (IQR 11.2) (range 20.8 to 54.8)
Silva 2010	None	None	n/a	n/a
van der Heijden 2015	31 (10 for exercise vs control)	1690	0% to 100% female; equally distributed across range	Mean 25 to 50 yr
Yamato 2015	10 (6 exercise vs minimal intervention (control))	478 (265 exercise vs control)	2 trials were all female, the others included both genders	Mean 38 yr (range 22 to 50)

CI: confidence interval; GP: general practitioner; IQR: interquartile range; OA: osteoarthritis; RCT: randomised controlled trial; ROM: range of motion; wk: week; yr: year.

#### Specificity of chronic pain condition of included reviews

Following abstract and full paper assessment, 21 reviews fulfilled the inclusion criteria: four in rheumatoid arthritis (Cramp 2013; Han 2004; Hurkmans 2009; Silva 2010), four in osteoarthritis (Bartels 2007; Fransen 2014; Fransen 2015; Regnaux 2015), three in fibromyalgia (Bidonde 2014; Busch 2007; Busch 2013), three in low back pain (Hayden 2005; Saragiotto 2016; Yamato 2015), two in intermittent claudication (Lane 2014; Lauret 2014), one in dysmenorrhoea (Brown 2010), one in mechanical neck disorder (Gross 2015a), one in spinal cord injury (Boldt 2014), one in postpolio syndrome (Koopman 2015), and one in patellofemoral pain (van der Heijden 2015). None of the included reviews assessed 'chronic pain' or 'chronic widespread pain' as a general term or specific condition.

The 21 included reviews were published by five different Cochrane Review groups: 11 from the Cochrane Musculoskeletal Group (Bartels 2007; Bidonde 2014; Busch 2007; Busch 2013; Cramp 2013; Fransen 2014; Fransen 2015; Han 2004; Hurkmans 2009; Regnaux 2015; Silva 2010); four from the Cochrane Neck and Back Group previously the Cochrane Back Group) (Gross 2015a; Hayden 2005; Saragiotto 2016; Yamato 2015); two from the Cochrane Peripheral Vascular Diseases Group (Lane 2014; Lauret 2014); one from the Cochrane Menstrual Disorders and Subfertility Group (Brown 2010); one from the Cochrane Injuries Group (Boldt 2014); one from the Cochrane Neuromuscular Group (Koopman 2015); and one from the Cochrane Bone, Joint and Muscle Trauma Group (van der Heijden 2015).

Protocols that may be included in updates of this overview focus on osteoarthritis (Østerås 2013 from the Cochrane Musculoskeletal Group), migraine (Brønfort 2015 from the Cochrane Pain, Palliative and Supportive Care Group), chronic low back pain (Hayden 2012 from the Cochrane Back Group), ankylosing spondylitis (Regnaux 2014 from the Cochrane Musculoskeletal Group)*,* and temporomandibular disorders (Craane 2006 from the Cochrane Oral Health Group).

#### Exercise and physical activity interventions implemented in the included reviews

Interventions assessed included: any specified style of land‐based exercise or physical activity such as one designed to improve strength, range of movement, aerobic capacity, or a combination of these (Boldt 2014; Busch 2007; Busch 2013; Cramp 2013; Fransen 2014; Fransen 2015; Gross 2015a; Hurkmans 2009; Koopman 2015; Regnaux 2015; van der Heijden 2015); a single style of land‐based exercise only (tai chi only: Han 2004, walking only: Lauret 2014, walking or jogging only: Brown 2010; Lane 2014, balance training only: Silva 2010, motor control exercise only: Saragiotto 2016, Pilates method only: Yamato 2015); any pool‐based or aquatic therapy (Bartels 2007; Bidonde 2014; Cramp 2013), or "any exercise therapy" (Hayden 2005).

##### Aquatic exercise

Any exercise performed in water. This can include swimming, though many studies will be referring to exercises performed vertically in the water (not horizontally), either using the water to support the body through the exercise, or as resistance against the body.

##### Range of motion and flexibility exercise

Can be performed in water or on land. The intention is to increase the range of motion around a joint through progressive stretching and mobilising of the muscles around and crossing the joint. For the purposes of this overview, we only included active movement where the movement was brought about by the participant, and not passively moved by an external force such as a therapist.

##### Aerobic exercise

Can be performed in water or on land. Exercise usually performed continuously to raise the heart rate and breathing rate for a prolonged period. Examples include walking, jogging, running, cycling, and swimming. Often presented as a percentage of the participant's heart rate max (HRmax) ‐ the highest heart rate reached when performing at their absolute maximum. Similarly it may be presented as a percentage of VO_2_max or VO_2_peak (a proportion of the maximum amount of oxygen the muscle can take up per minute), or as an absolute value (mL/kg/minute).

##### Strength/resistance exercise

Can be performed in water or on land. Exercise performed against a progressive resistance with the intention of improving muscle strength, muscle endurance, muscle power, or a combination of these. Resistance can come from fixed or free weights, elastic bands, body weight (against gravity), and water resistance. It may also involve static or isometric strength (holding a position or weight without moving against it). Often presented as a percentage of the participant's one repetition maximum (1‐RM) ‐ the maximum weight they can lift/move if they only have to do it once.

##### Motor control exercise

Can be performed in water or on land. Exercise to bring about activation of the deep trunk muscles, targeting the restoration of control and co‐ordination of these 'core muscles' (Saragiotto 2016).

##### Balance (proprioceptive) training

Can be performed in water or on land (water may be used initially for support). Exercise emphasises the maintenance of balance during visual and perturbation challenges with eyes open or closed, range of motion, and maintaining stability over reduced areas of support and unstable surface (Silva 2010), that is improving balance in increasingly unstable situations.

##### Tai chi

An ancient Chinese discipline developed from martial arts, involving a continuous series of very controlled (and usually slow) movements designed to improve physical and mental wellbeing.

##### Yoga

Arising out of Hindu philosophy. Exercise includes breath control, simple meditation, and the adoption of specific bodily postures. It is widely practised for health, relaxation, and control (physically and mentally). Incorporates stretching and flexibility training with isometric strength training (holding certain poses, with no movement against a resistance).

##### Pilates

Developed by Joseph Pilates in the 20th Century, it is a system of exercises (often using special apparatus) designed to improve physical strength, flexibility, and posture, and enhance mental awareness.

#### Duration and dose (frequency/intensity) of the exercise and physical activity interventions

A detailed breakdown of each review can be seen in Table 5.

**5:** Dose and duration of exercise interventions in included reviews

**Review**	**Duration**	**Frequency (sessions per day/wk/month)**	**Intensity**	**Duration (per session)**	**Other description**
Bartels 2007	Not reported	Not reported	"Muscle maintenance" and "range of motion"	Not reported	No minimum requirement for inclusion. Actual intervention only reported by 2 of 6 included studies.
Bidonde 2014	17 wk (range 4 to 32)	1 to 4/wk	Very light (< 57% HRmax) to vigorous (95% HRmax), self‐selected, and not specified	45 minutes (range 30 to 70)	No minimum requirement for inclusion. None of the studies met the ACSM exercise guidelines specified for aerobic or strength training. Only 1 study met the ACSM guidelines for flexibility training.
Boldt 2014	12 wk to 9 months	2/day to 2/wk	Not reported	Reported for 1 study only (90 to 120 minutes)	No minimum requirement for inclusion. Stretching and strengthening exercises aimed at mobilising painful shoulder joint.
Brown 2010	≥ 12 wk	3/wk	70% to 85% HRR	1 hour	No minimum requirement for inclusion.
Busch 2007	3 wk to 6 months	1 to 5/wk	Not reported	Not reported	No minimum requirement for inclusion. Assessed as whether they "met ACSM recommendations."
Busch 2013	8 to 21 wk (median 16 wk)	≥ 2/wk	> 4/10 RPE rating progressing to 70% to 80% 1RM	40 to 90 minutes	Assessed as whether they "met ACSM recommendations."
Cramp 2013	6 wk (when reported)	2/wk	"Low impact", "moderate", and 70% HRmax	1 to 1.5 hours, when reported	No minimum requirement for inclusion.
Fransen 2014	6 to 12 wk (median 8)	1 to 3/wk	"Low intensity" to "max effort"	30 to 60 minutes	No minimum requirement for inclusion. Intensity only reported in 2 of 10 studies.
Fransen 2015	single session to 30 months	1 to 5/wk	"Moderate to moderately high intensity"	15 to 60 minutes	No minimum requirement for inclusion. Varied in dose and duration.
Gross 2015a	2 wk to 3 months	5/wk to every 15 minutes/day	Low intensity	2 to 20 minutes	‐
Han 2004	8 to 10 wk (when reported)	1 to 7/wk (median 1/wk)	Tai chi = low intensity	1 to 1.5 hours	No minimum requirement for inclusion.
Hayden 2005	Not reported	Not reported	Not reported	Not reported	No minimum requirement for inclusion. Could not extract actual data.
Hurkmans 2009	≥ 6 wk	2/wk	Aerobic: ≥ 55% HRmax increasing to 85% HRmax strength: start 30% 1RM increasing to 80% 1RM	20 minutes	‐
Koopman 2015	4 to 12 wk	Daily to 3/wk	Reported in 1 study: 50% to 70% MVC	45 minutes	No minimum requirement for inclusion. 1 study: supervised progressive resistance training consisting of 3 sets of 8 isometric contractions of the thumb muscles. 1 study: combination of individual and group therapy with daily treatment in a swimming pool (45 minutes), physiotherapy, individually adapted training programme.
Lane 2014	3 to 12 months	≥ 2/wk	"Variable"	˜ 60 minutes	No minimum requirement for inclusion.
Lauret 2014	≥ 6 wk	≥ 2/wk	Not reported	Not reported	No minimum requirement for inclusion. Must be supervised.
Regnaux 2015	8 wk	3/wk	Compared high vs low intensity vs control	30 to 50 minutes	Every 2 wk 1RM was retested and increased by 5% as tolerated in each group. Supervision: an experienced therapist. 3 arms (n=34 per arm): high intensity, low intensity, control (no exercise).
Saragiotto 2016	20 days to 12 wk (median 8 wk (IQR 2.0))	1 to 5/wk (median 12 sessions (IQR 6.0))	Not reported	20 to 90 minutes (median 45 (IQR 30) minutes)	MCE is usually delivered in 1:1 supervised treatment sessions, and sometimes involves ultrasound imaging, the use of pressure biofeedback units or palpation to provide feedback on the activation of trunk muscles.
Silva 2010	≥ 6 wk	2/wk	Balance training only	≥ 30 minutes	No studies found.
van der Heijden 2015	3 to 16 wk	2/wk to daily	Not reported	Not reported	No minimum requirement for inclusion. Assessed by duration (< or > 3 months), frequency (several times, or once a week), medium (land or water), etc.
Yamato 2015	10 to 90 days (mostly 8 wk)	2/wk (mean session number 15.3, range 6 to 30)	Not reported	1 hour	No minimum requirement for inclusion. Must be supervised (for the Pilates technique).

1RM: one repetition maximum; ACSM: American College of Sport Medicine; HRmax: maximum heart rate; HRR: heart rate reserve, IQR: interquartile range; MCE: motor control exercise; MVC: maximum voluntary contraction; RPE: rating of perceived exertion; wk: week.

##### Duration of intervention

Interventions assessed by the included reviews varied in length from a single session (Fransen 2015) to 30 months (Fransen 2015). Only five reviews enforced a minimum intervention period to reduce risk of bias, and were able to attribute any effects to the intervention (Brown 2010; Busch 2013; Gross 2015a; Hurkmans 2009; Silva 2010).

##### Frequency

There was large variation in the exercise or physical activity intervention being implemented, ranging from just once a week (Bidonde 2014; Busch 2007; Fransen 2014; Fransen 2015; Han 2004; Saragiotto 2016), to twice a day (Boldt 2014), and some performing a short series of exercises (two‐minute duration) every 15 minutes during the day (Gross 2015a). However, when reported, most included studies in the reviews implemented the programme twice a week (or stated at least twice a week).

##### Intensity

Few studies quantified the intensity of each session. Baseline intensity was often accepted as low/moderate, with the aim to progress over the intervention period to 70% to 85% of HRmax or heart rate reserve (HRR) for aerobic interventions (Brown 2010; Cramp 2013; Hurkmans 2009), 70% to 80% of an individual's 1‐RM, or 50% to 70% maximum voluntary contraction (Koopman 2015) in strength/resistance training programmes (Busch 2013; Hurkmans 2009). In other reviews, intensity was described more loosely as "variable" or "low intensity (very light) to maximum effort (vigorous)" (Bidonde 2014; Fransen 2014; Lane 2014; Regnaux 2015), "low intensity" (Fransen 2014; Gross 2015a; Han 2004; Silva 2010), or "moderate or moderate‐to‐high" (Cramp 2013; Fransen 2015).

##### Duration (per session)

Individual sessions varied in length from two minutes (Gross 2015a), to 90 minutes (Busch 2013; Cramp 2013; Han 2004) or 120 minutes (Boldt 2014), but mostly situated around 45 to 60 minutes. However, it is important to note that the shorter sessions were often performed more regularly than longer sessions. With more information it would have been possible to calculate total volume of exercise or physical activity (session duration × frequency per week × number of weeks), for a more accurate and detailed analysis.

#### Intervention specificity for chronic pain in the included reviews

The focus of this overview was exercise versus no‐exercise interventions with the intention of answering the original question: is exercise beneficial, detrimental, or ineffective for people with chronic pain when compared to inactivity? Two of the 21 reviews did not include/locate any studies that examined simply exercise versus no exercise (Lauret 2014; Silva 2010). However, many of the included reviews compared varying exercise modality, duration, intensity, and frequency. The "no‐exercise" intervention referred to the control group where there was a minimal intervention (such as sham exercise or education) or wait‐list control/no treatment (see Table 3 for more information on control group activity).

#### Time points reported

Four of the 19 reviews that reported data, reported results at a single time point only ('post‐intervention': Bidonde 2014; Busch 2007; Cramp 2013; Han 2004). Reviews also analysed outcome measures immediately post‐intervention and at one or more follow‐up points. Each review defined short‐, intermediate‐, and long‐term follow‐up according to their own assessment, so when the time period was not mentioned explicitly, we grouped the reviews according to the review authors' own classification only, and where a time period (weeks, month, years) was explicitly listed but not defined by the authors, we grouped them as short‐term (follow‐up as under six months), intermediate‐term (six to 12 months), and long‐term (longer than 12 months): short‐term: Busch 2013; Fransen 2014; Fransen 2015; Gross 2015a; Hayden 2005; Lane 2014; Regnaux 2015; Saragiotto 2016; intermediate‐term: Bartels 2007; Fransen 2015; Gross 2015a; Hayden 2005; Lane 2014; Regnaux 2015; Saragiotto 2016; long‐term: Gross 2015a; Hayden 2005; Regnaux 2015; Saragiotto 2016. Five reviews did not report "post‐intervention" but at short‐term, mid/intermediate‐term, and long‐term postrandomisation (short, mid, and long term: Boldt 2014; short and intermediate term: Koopman 2015; Yamato 2015; short and long‐term: Hurkmans 2009; van der Heijden 2015). One review assessed participants in an ongoing fashion "over three menstrual cycles" (Brown 2010).

##### Long‐term follow‐up

Of the seven reviews claiming to report "long term" follow‐up, one classed long‐term as longer than six weeks (intermediate term as one to six weeks' follow‐up) (Boldt 2014). The remaining six reviews defined long‐term follow up as over 12 months (one year) post‐intervention (Gross 2015a; Hayden 2005; Hurkmans 2009; Regnaux 2015; Saragiotto 2016; van der Heijden 2015).

### Methodological quality of included reviews

#### AMSTAR quality assessment of included reviews

No review achieved a perfect score of 11/11, though five achieved 10/11 (Boldt 2014; Busch 2013; Hayden 2005; Koopman 2015; Regnaux 2015) and eight scored 9/11 (Cramp 2013; Gross 2015a; Hurkmans 2009; Lane 2014; Lauret 2014; Saragiotto 2016; van der Heijden 2015; Yamato 2015). The lowest score was 6/11 (Silva 2010) though five categories were not applicable (n/a) due to there being no included studies. Quality assessment results for each individual review are presented in Table 6.

**6:** Methodological quality of included reviews using the AMSTAR tool

**Review**	**Criteria**											**Total "Y"**	**Total "N"**	**Total "n/a"**
	**1**	**2**	**3**	**4**	**5**	**6**	**7**	**8**	**9**	**10**	**11**			
Bartels 2007	Y	Y	Y	Y	Y	N	Y	Y	Y	N	N	8	3	‐
Bidonde 2014	N	Y	Y	Y	Y	Y	Y	Y	Y	N	N	8	3	‐
Boldt 2014	Y	Y	Y	Y	Y	Y	Y	Y	Y	Y	N	10	1	‐
Brown 2010	Y	Y	Y	N	Y	Y	Y	Y	n/a	N	N	7	3	1
Busch 2007	Y	Y	Y	N	Y	Y	Y	Y	Y	N	N	8	3	‐
Busch 2013	Y	Y	Y	Y	Y	Y	Y	Y	Y	Y	N	10	1	‐
Cramp 2013	Y	Y	Y	Y	Y	N	Y	Y	Y	Y	N	9	2	‐
Fransen 2014	Y	Y	Y	Y	Y	N	Y	Y	Y	N	N	8	3	‐
Fransen 2015	Y	Y	Y	Y	Y	N	Y	Y	Y	N	N	8	3	‐
Gross 2015a	Y	Y	Y	Y	Y	N	Y	Y	Y	Y	N	9	2	‐
Han 2004	Y	Y	Y	Y	Y	N	Y	Y	N	N	N	7	4	‐
Hayden 2005	Y	Y	Y	Y	Y	N	Y	Y	Y	Y	Y	10	2	‐
Hurkmans 2009	Y	Y	Y	Y	Y	Y	Y	Y	Y	N	N	9	2	‐
Koopman 2015	Y	Y	Y	Y	Y	Y	Y	Y	Y	N	Y	10	1	‐
Lane 2014	Y	Y	Y	Y	Y	Y	Y	Y	N	Y	N	9	2	‐
Lauret 2014	Y	Y	Y	Y	Y	N	Y	Y	Y	Y	N	9	2	‐
Regnaux 2015	Y	Y	Y	Y	Y	Y	Y	Y	Y	Y	N	10	1	‐
Saragiotto 2016	Y	Y	Y	Y	Y	N	Y	Y	Y	Y	N	9	2	‐
Silva 2010	Y	Y	Y	Y	Y	n/a	n/a	n/a	n/a	n/a	Y	6	0	5
van der Heijden 2015	Y	Y	Y	Y	Y	Y	Y	Y	Y	N	N	9	2	‐
Yamato 2015	Y	Y	Y	Y	Y	N	Y	Y	Y	Y	N	9	2	‐
**Total "Y"**	**20**	**21**	**21**	**19**	**21**	**10**	**20**	**20**	**17**	**10**	**3**	‐	‐	‐
**Total "N"**	**1**	**‐**	**‐**	**2**	**‐**	**10**	**‐**	**‐**	**2**	**10**	**18**	‐	‐	‐
**Total "n/a"**	**‐**	**‐**	**‐**	**‐**	**‐**	**1**	**1**	**1**	**2**	**1**	**‐**	‐	‐	‐

N: no; n/a: not applicable; Y: yes; out of maximum summative score of 11. Following arbitration, the authors removed the response "cannot answer" due to no responses as such.

All reviews except one (Bidonde 2014) fulfilled the basic criteria (questions one to three of Table 1); to follow an 'a priori' design as Cochrane implements a system of protocol publication before undertaking the full reviews, where it also specifies dual study selection and data extraction from a comprehensive literature search. One review did not fulfil the 'a priori' design as this was an update and separation from a broader review series, and so the criteria had not been explicitly listed prior to publication for this specific title (Bidonde 2014).

Criteria which scored badly using the AMSTAR tool were characteristics of included studies (question six of Table 1), reporting of publication bias (question 10 of Table 1), and conflict of interest declarations (question 11 of Table 1).

- Included study characteristics were limited, often reporting the "inclusion criteria" used to recruit participants in the study instead of the characteristics of actual included participants, and excluding information such as participants' age, gender split, ethnicity, and disease status.

- Assessment of publication bias was omitted entirely in five reviews (Bartels 2007; Fransen 2014; Fransen 2015; Han 2004; Hurkmans 2009), and when it was assessed, it was reported using only a simple statement (with no test values, analyses used, or diagrams to demonstrate the result; Busch 2007; Koopman 2015). Two reviews mentioned in the methods as planned analyses, though was not mentioned again (Brown 2010; van der Heijden 2015), and a third review mentioned it in the methods, but appeared to use it interchangeably with reporting bias causing great confusion (Bidonde 2014).

- Conflicts of interest were sufficiently reported in only three out of 21 of the included reviews (Hayden 2005; Koopman 2015; Silva 2010). In the remaining reviews, a cursory statement was commonly made regarding the review authors' conflicts of interests, however, fulfilling the AMSTAR criteria also requires a statement to be made regarding any conflict of interest for any of the included studies.

#### Risk of bias in included reviews

The original review authors assessed risk of bias (see Table 7). The table shows the number of studies assessed as low risk of bias only, and excluded those that were assessed as unclear or high risk of bias.

**7:** Risk of bias ‐ studies assessed as low risk of bias

**Review**	**Number of studies in assessment**	**Selection bias**		**Performance bias**	**Detection bias**	**Attrition bias**	**Reporting bias**	**Other bias**	
		**Random sequence generation (studies)**	**Allocation concealment (studies)**	**Blinding of participants and personnel (studies)**	**Blinding of outcome assessment (studies)**	**Incomplete outcome data (studies)**	**Selective reporting (studies)**	**Sample size**	**Other biases (studies)**
Bartels 2007	6	Not reported	3	Not reported	2	3	Not reported	2, n > 100 per arm	‐
Bidonde 2014	9	5	3	2	8	8	5	1, n > 50 per arm	7
Boldt 2014	3	1	1	0	1	2	3	0	1
Brown 2010	1	0	0	0	0	1	1	1, n > 50 per arm	‐
Busch 2007	34	17	10	8	20	Unclear	32	5, n > 50 per arm	‐
Busch 2013	5	4	2	1	2	5	3	0, n > 50 per arm	‐
Cramp 2013	7	5	2	0	Not reported	6	4		1
Fransen 2014	10	8	7	0	0	7	4	1, n > 50 per arm	7
Fransen 2015	54	40	22	3	4	29	10	5, total n > 200	
Gross 2015a	16	8	8	1	0	11	0	0	11
Han 2004	4	2	0	0	0	0	Not reported	0	
Hayden 2005	43	27	22	Not reported	12	29	Not reported	10, total n > 100 + 5, total n > 200	‐
Hurkmans 2009	8	8	1	‐	4	5	‐	1, total n > 200	1
Koopman 2015	2	1	0	0	0	0	0	0	1
Lane 2014	30	16	14	30	7	19	29	3, total n > 100	
Lauret 2014	5	4	2	5	3	4	5	1, total n > 100	4
Regnaux 2015	1	1	0	0	1	0	0	1, total n > 100	1
Saragiotto 2016	7	5	4	1	1	2	7	1, total n > 100 + 1, total n > 200	7
Silva 2010	0	n/a	n/a	n/a	n/a	n/a	n/a	n/a	n/a
van der Heijden 2015	10	8	6	0	0	6	9	2, total n > 100	10
Yamato 2015	9	5	5	2	7	7	9	0	9
**Studies with low risk of bias (number)**	**264**	**165**	**112**	**53**	**72**	**144**	**121**	**total n > 100: 26 total n > 200: 15 total n > 400: 0**	**71**
**Studies with low risk of bias (percentage)**	**‐**	**63%**	**42%**	**20%**	**27%**	**55%**	**46%**	**total n > 100: 10% total n > 200: 6% total n > 400: 0%**	**27%**

n: number of participants, n/a: not applicable.

##### Selection bias (randomisation and allocation concealment)

Selection bias had the largest proportion of included studies with low risk of bias (63% and 42% of studies adequately undertaking and reporting the methods used).

##### Performance and detection bias (blinding participants, personnel, outcome assessors)

With any exercise or physical activity intervention it is very difficult to blind both participants and personnel to the allocation, though some studies included in reviews attempted to by offering sham exercise.

Due to the difficulty of blinding participants to their group allocation, review authors assessed the risk of bias in different ways, which may cause confusion: whereas the majority declared this lack of possible blinding to be high risk of bias or unclear, two reviews labelled such cases as low risk of bias in order not to exclude these studies unnecessarily from their analysis (Lane 2014;Lauret 2014). Without these two reviews, only a small percentage (7.8% or 18/229) of the included studies would have scored low risk of performance bias (blinding of participants and personnel), but by including them (all 35 studies from those two reviews assessed as low risk of bias) the overall proportion of studies assessed as having low risk of bias was closer to 20% (53/264).

##### Attrition (incomplete outcome data, withdrawals/dropouts)

About 55% (144/264) of the studies included in these reviews showed low risk of bias.

##### Reporting bias (selective reporting)

Reporting bias was classed as low risk in only 46% of included studies. However, it is important to note this was not due to the remainder having high risk of bias, but instead 'unclear', as trial protocols were not always published or accessible to the review authors to accurately assess/interpret.

##### Study/sample/group size

Sample size was not always included within the risk of bias assessment. It was therefore extracted directly from each review's table of included study characteristics by a single overview author (LG), and assessed as being low risk of bias when there was a minimum of 50 participants per arm, or 100 in total. Numbers were then separated for the proportion of studies with greater than 100 participants per arm (or 200 in total), and 200 participants per arm (or 400 in total), as this could then be considered higher tiered evidence.

Only 26 out of 264 included studies (10%) across the 21 reviews reported over 100 participants in total (or 50 per arm), a further 6% (15/264) included over 200 participants per arm. The remaining 223 studies (84%) had fewer than 50 participants per arm (or sample size was not reported), often not reaching 50 in total.

##### Other bias

The format for reporting bias has changed, and therefore some earlier reviews (that are yet to be updated) did not assess bias using the same format. Others reported additional criteria as 'other bias' including the similarity of baseline characteristics, and similarity of timing points.

#### Interpretation of results/conclusions by original review authors

For conclusions made by the original review authors, see Table 8. We assessed whether these conclusions/interpretations of the results accurately reflected the information provided within the review, and if any further information should have been included. This final assessment of the review is an important stage in determining any author bias within the review process, as many readers, funders, and policy makers will focus on the author conclusions without a full appraisal of the actual presented data.

**8:** Interpretation of results by original review authors

**Review**	**Review authors' conclusions**	**Overview authors' assessment of conclusions**
Bartels 2007	"Aquatic exercise has some short‐term beneficial effects on the condition of OA patients with hip or knee OA or both. The controlled and randomised studies in this area are still too few to give further recommendations on how to use this therapy... No long‐term effects have been found."	Appropriate conclusions based on available data. No mention of quality/risk of bias in conclusions, though found to be high quality in results section.
Bidonde 2014	"Low to moderate quality evidence relative to control suggests that aquatic training is beneficial for improving wellness, symptoms, and fitness in adults with fibromyalgia. Very low to low quality evidence suggests that there are benefits of aquatic and land‐based exercise, except in muscle strength (very low quality evidence favoring land). No serious adverse effects were reported."	Appropriate conclusions based on available data.
Boldt 2014	"Evidence is insufficient to suggest that non‐pharmacological treatments are effective in reducing chronic pain in people living with SCI. The benefits and harms of commonly used non‐pharmacological pain treatments should be investigated in randomised controlled trials with adequate sample size and study methodology"	Appropriate conclusions based on available data.
Brown 2010	"There is a lack of available evidence to support the use of exercise in the alleviation of symptoms associated with dysmenorrhoea. The limited evidence implies that there are no adverse effects associated with exercise."	Review authors should not have commented on lack of adverse events as this was not reported in the included study. The comment on lack of adverse events contravened present Cochrane guidance.
Busch 2007	"There is moderate quality evidence that short‐term aerobic training (at the intensity recommended for increases in cardiorespiratory fitness) produces important benefits in people with FM in global outcome measures, physical function, and possibly pain and tender points. There is limited evidence that strength training improves a number of outcomes including pain, global wellbeing, physical function, tender points and depression. There is insufficient evidence regarding the effects of flexibility exercise. Adherence to many of the aerobic exercise interventions described in the included studies was poor."	Appropriate conclusions based on available data.
Busch 2013	"We have found evidence in outcomes representing wellness, symptoms, and physical fitness favoring resistance training over usual treatment and over flexibility exercise, and favoring aerobic training over resistance training. Despite large effect sizes for many outcomes, the evidence has been decreased to low quality based on small sample sizes, small number of randomized clinical trials (RCTs), and the problems with description of study methods in some of the included studies."	Appropriate conclusions based on available data.
Cramp 2013	"There is some evidence that physical activity interventions ... may help to reduce fatigue in RA. However, the optimal parameters and components of these interventions are not yet established."	Appropriate conclusions based on available data. However, no mention of quality/risk of bias of studies in conclusion despite low/unclear quality score in results and discussion sections. No conclusions about effect on pain (insufficient data).
Fransen 2014	"There is currently high‐level evidence that land‐based exercise will reduce hip pain, and improve physical function, among people with symptomatic hip osteoarthritis."	Evidence was good quality though sample sizes were often small (i.e. it is debatable if this was high level evidence as claimed by authors). Agree that results demonstrate small but significant benefit from intervention.
Fransen 2015	"High‐quality evidence suggests that land‐based therapeutic exercise provides benefit in terms of reduced knee pain and quality of life and moderate‐quality evidence of improved physical function among people with knee OA… Despite the lack of blinding we did not downgrade the quality of evidence for risk of performance or detection bias."	Appropriate conclusions based on available data. May have been generous with quality assessment but this was stated in conclusions for transparency.
Gross 2015a	"…there is still no high quality evidence and uncertainty about the effectiveness of exercise for neck pain… Moderate quality evidence supports the use specific strengthening exercises as a part of routine practice … Moderate quality evidence supports the use of strengthening exercises, combined with endurance or stretching exercises may also yield similar beneficial results. However, low quality evidence notes when only stretching or only endurance type exercises … there may be minimal beneficial effects for both neck pain and function."	Appropriate conclusions based on available data.
Han 2004	"Tai chi appears to have no detrimental effects on the disease activity of RA in terms of swollen/tender joints and activities of daily living…tai chi appears to be safe, since only 1 participant out of 121 withdrew due to adverse effects and withdrawals were greater in the control groups than the tai chi groups."	Appropriate conclusions based on available data. However, no mention of quality/risk of bias in conclusion despite very low quality score in results section.
Hayden 2005	"Evidence from randomized controlled trials demonstrates that exercise therapy is effective at reducing pain and functional limitations in the treatment of chronic low‐back pain, though cautious interpretation is required due to limitations in this literature."	Appropriate conclusions based on available data. However, no mention of quality/risk of bias of studies in conclusion despite low quality score in results and discussion sections.
Hurkmans 2009	"Short‐term, land‐based dynamic exercise programs have a positive effect on aerobic capacity (aerobic capacity training whether or not combined with muscle strength training) and muscle strength (aerobic capacity training combined with muscle strength training) immediately after the intervention, but not after a follow‐up period. Short‐term, water‐based dynamic exercise programs have a positive effect on functional ability and aerobic capacity directly after the intervention but it is unknown whether these effects are maintained after follow‐up. Long‐term, land‐based dynamic exercise programs (aerobic capacity and muscle strength training) have a positive effect on functional ability, aerobic capacity, and muscle strength immediately after the intervention but it is unknown whether these effects are maintained after follow‐up... Based on the evidence, aerobic capacity training combined with muscle strength training is recommended for routine practice in patients with RA."	Appropriate conclusions based on available data. However, no mention of quality/risk of bias of studies in conclusion. No conclusions regarding pain severity.
Koopman 2015	"Data from two single trials suggested that muscle strengthening of thumb muscles (very low‐quality evidence) ... are safe and beneficial for improving muscle strength ... with unknown effects on activity limitations." "We found evidence varying from very low quality to high quality that ... rehabilitation in a warm or cold climate are not beneficial in PPS." "Due to a lack of good‐quality data and randomised studies, it was impossible to draw definitive conclusions about the effectiveness of interventions in people with PPS."	Appropriate conclusions based on available data.
Lane 2014	"… Exercise therapy should play an important part in the care of selected patients with intermittent claudication, to improve walking times and distances. Effects were demonstrated following three months of supervised exercise although some programmes lasted over one year."	Appropriate conclusions based on available data. However, no mention of quality/risk of bias of studies in conclusion. No conclusions regarding pain severity.
Lauret 2014	"There was no clear evidence of differences between supervised walking exercise and alternative exercise modes in improving the maximum and pain‐free walking distance of patients with intermittent claudication…. The results indicate that alternative exercise modes may be useful when supervised walking exercise is not an option for the patient."	Appropriate conclusions based on available data. However, no mention of quality/risk of bias of studies in conclusion (in discussion).
Regnaux 2015	"We found very low‐ to low‐quality evidence for no important clinical benefit of high‐intensity compared to low‐intensity exercise programs in improving pain and physical function in the short term.... The included studies did not provide any justification for the levels of intensity of exercise programs. No authors reported evidence for the minimal and maximal intensity that could be delivered."	Appropriate conclusions based on available data. This overview has only used one study of the six included as it alone included a control group, for which we could not extract data as the control comparison was not used in the analysis by the review authors.
Saragiotto 2016	"There is very low to moderate quality evidence that MCE has a clinically important effect compared with a minimal intervention for chronic low back pain... As MCE appears to be a safe form of exercise and none of the other types of exercise stands out, the choice of exercise for chronic low back pain should depend on patient or therapist preferences, therapist training, costs and safety."	Appropriate conclusions based on available data.
Silva 2010	"We were not able to provide any evidence to support the application of balance exercises (proprioceptive training) alone in patients with RA."	Appropriate conclusions based on available data (no included studies).
van der Heijden 2015	"This review has found very low quality but consistent evidence that exercise therapy for patellofemoral pain syndrome (PFPS) may result in clinically important reduction in pain and improvement in functional ability."	No subgroup analysis to differentiate between acute, subacute, and chronic pain made it difficult to extract appropriate data for this review.
Yamato 2015	"No definite conclusions or recommendations can be made as we did not find any high quality evidence for any of the treatment comparisons, outcomes or follow‐up periods investigated. However, there is low to moderate quality evidence that Pilates is more effective than minimal intervention in the short and intermediate term as the benefits were consistent for pain intensity and disability, with most of the effect sizes being considered medium."	Appropriate conclusions based on available data. There was no subgroup analysis to differentiate between acute, subacute, and chronic pain made it difficult to extract appropriate data for this review (one included study had subacute back pain (> 6 weeks), all others were chronic back pain (> 12 weeks)) but results are presented altogether as chronic pain.

FM: fibromyalgia; MCE: motor control exercise; OA: osteoarthritis; PPS: postpolio syndrome; RA: rheumatoid arthritis; SCI: spinal cord injury.

Eleven of the 21 reviews reported appropriate conclusions based on the data available in the context of the quality of evidence (Bidonde 2014; Boldt 2014; Busch 2007; Busch 2013; Fransen 2015; Gross 2015a; Koopman 2015; Regnaux 2015; Saragiotto 2016; Silva 2010; Yamato 2015); five reviews had appropriate conclusions, did not mention quality of the evidence in the conclusion, but did discuss it in detail earlier in the review (Bartels 2007; Cramp 2013; Han 2004; Hayden 2005; Lauret 2014); two reviews had appropriate conclusions but had only limited discussion of quality or did not adequately consider the quality of the evidence in the interpretation of the results (Hurkmans 2009; Lane 2014); and three reviews needed further comment as the strength of the conclusions were not appropriate based on the available data (Brown 2010; Fransen 2014), or we were unable to agree with their interpretation due to difficulty in extracting the data (van der Heijden 2015).

### Effect of interventions

We have interpreted results using data reported in the reviews, and did not return to the original studies. Where data have been reported as MDs or as an absolute or relative change score we have used the appropriate scales (where possible) to determine whether this was clinically significant. When data have only been presented as SMD, with or without 95% confidence intervals (CI), with or without level of significance (P value), we have cautiously used the interpretation by Cohen 1988 who defined effect size using the SMD as small (SMD 0.2 to 0.5), moderate (SMD 0.5 to 0.8), or large (SMD greater than 0.8).

For the purposes of clarity, we have used the term 'intervention' to refer to the exercise or physical activity intervention, and 'control' to refer to the included comparison group which did not involve any exercise or physical activity element.

#### Primary outcome

##### Self‐reported pain (severity)

Part of the inclusion criteria for this overview was for pain severity to be listed as an outcome measure.

Two of the 21 reviews did not include/identify any studies that examined intervention versus control (Lauret 2014; Silva 2010). Of the remaining reviews that did report studies examining intervention versus control (no physical activity or exercise, or minimal intervention), two did not report pain as an absolute or relative score of severity, intensity, or change as a result of the intervention (Brown 2010; Han 2004), and one review assessed pain‐free time and distance during exercise (they did not assess pain using a mean/usual pain scale; Lane 2014). We could not extract relevant data for one review as they compared two different exercise interventions and a control but did not report the data compared to the control (Regnaux 2015).

The remaining 15 reviews reported a mean or usual pain score for exercise (intervention) and no‐exercise (control) groups (Bartels 2007; Bidonde 2014; Boldt 2014; Busch 2007; Busch 2013; Cramp 2013; Fransen 2014; Fransen 2015; Gross 2015a; Hayden 2005; Hurkmans 2009; Koopman 2015; Saragiotto 2016; van der Heijden 2015; Yamato 2015).

###### Reported baseline pain score

Of the 15 reviews that were able to assess pain (Table 9), only three reviews reported actual baseline pain scores (Bidonde 2014; Boldt 2014; Hayden 2005). Three reviews reported change data (Bartels 2007; Busch 2007; Busch 2013), but we were able to use control group baseline and earliest control group scores as assumed or approximate baseline measures for the intervention groups in nine reviews (Bartels 2007; Busch 2007; Fransen 2014; Fransen 2015; Gross 2015a; Koopman 2015; Saragiotto 2016; van der Heijden 2015; Yamato 2015). Overall, only three reviews that assessed pain did not provide baseline or control group scores for comparison (Busch 2013; Cramp 2013; Hurkmans 2009).

**9:** Pain severity

**Review**	**Number of trials (and participants) assessing 'pain severity'**	**Baseline pain score**	**Post‐intervention reported result or change data (or if only one data point reported in review)**	**Follow‐up**	**Overall comment/statement**
Bartels 2007 (osteoarthritis)	Hip + knee OA: Post‐intervention: 4 (638) Follow‐up: 1 (310) Hip only: follow‐up: 1 (17) Knee only: post‐intervention: 1 (46)	Control baseline: Hip + knee OA WOMAC 0 to 20 (2 studies): 9.10 (SD 3.14) VAS 0 to 100 (1 study): 55.3 (SD 24.6) HAQ 0 to 3 (1 study): 1.05 (SD 0.61) Hip only VAS 0 to 100 (1 study): 56 (SD 21.89) Knee only VAS 0 to 10 (1 study): 5.6 (SD 1.4)	Hip + knee OA A minor effect of a 3% absolute reduction (0.6 fewer points on WOMAC 0 to 20 scale) and 6.6% relative reduction SMD 0.19 (95% CI 0.04 to 0.35) (P = 0.02) Knee only SMD 0.86 (95% CI 0.25 to 1.47) (P = 0.005) Absolute difference 12% (1.2 fewer points on a 0 to 10 scale) Relative change 22% improvement	Hip + knee OA Follow‐up at 6 months: SMD 0.11 (95% CI ‐0.12 to 0.33) (ns) No difference Hip only SMD 1.00 (95% CI ‐0.04 to 2.04) (P = 0.06, ns)	Statistically significant post‐intervention in hip + knee OA group, but not clinically significant. Knee‐only OA had moderate to large effect size (statistically significant) immediately post‐intervention.
Bidonde 2014 (fibromyalgia)	Post‐intervention: 7 (382)	Weighted mean score at baseline (all participants): 69.59 median value for pain was 70.9 in studies comparing aquatic training to control	On 100‐point scale: MD ‐6.59 (95% CI ‐10.71 to ‐2.48) SMD ‐0.53 (95% CI ‐0.76 to ‐0.31) Absolute difference ‐7% (95% CI ‐11 to ‐3) NNTB 5 (95% CI 3 to 8)	3 studies at 12, 48, or 52 weeks' post‐intervention could not be combined. 2 studies showed SMD favouring intervention at follow‐up.	"We found a moderate effect favouring the aquatic exercise training for pain" …"similar improvements in pain in the low pain groups (SMD ‐0.60, 95% CI ‐0.98 to ‐0.23) and in the high pain groups (SMD ‐0.57, 95% CI ‐1.11 to ‐0.03)." Among the major wellness outcomes, none of the outcomes met the threshold for clinically relevant differences (15%).
Boldt 2014 (spinal cord injury)	Post‐intervention: 3 (149)	WUSPI score 22.6 (exercise group) to 11.05 (control group) in 1 group at baseline Not reported for 2 studies	WUSPI change score: Exercise group: ‐7.7 (SD 19.01) Control group: 12.8 (SD 12.74) SF‐36 (pain experience): ‐1.9 (95% CI ‐3.4 to ‐0.4) favoured exercise (P = 0.01) VAS (0 to 10): MD ‐2.8 (95% CI ‐3.77 to ‐1.83) favoured exercise (P < 0.00001)	1 study at 4 weeks: VAS (0 to 10): ‐2.50 (95% CI ‐3.48 to ‐1.52) (P < 0.00001) WUSPI: ‐26.40 (95% CI ‐37.62 to ‐15.18favoured exercise (P < 0.00001)	"All three studies were fraught with high overall risk of bias. In particular, the comparison with 'no treatment' or waiting lists as control interventions likely leads to an overestimation of the effectiveness of the exercise programmes provided in these studies. Consequently, no conclusion on their effectiveness can be drawn."
Busch 2007 (fibromyalgia)	Strength training: 1 (21) Aerobic training: 3 (183)	Control baseline: Aerobic: 6.1/10 (VAS) (SD 1.97) Strength: 35/100 (VAS) (SD 19)	Aerobic training: SMD 0.65 (95% CI ‐0.09 to 1.39) (ns) Weighted absolute change 13% (1.3 cm lower on 10‐cm scale) Relative change 21% Strength training: SMD 3.00 (95% CI 1.68 to 4.32) (ns) Weighted absolute change 49% (49 points lower on 100‐point scale) Relative change 140%, NNTB 2	n/a	">30% improvement was seen in the strength training group as compared to an untreated control group in pain." Aerobic training led to an improvement of 1.3/10.
Busch 2013 (fibromyalgia)	Post‐intervention: 2 (81) Follow‐up at 8 weeks, 16 weeks, 28 weeks: 1 (60)	Not reported ‐ change data only	Change score on VAS (in cm): MD ‐3.30 (95% CI ‐6.35 to ‐0.26) (P = 0.03) SMD ‐1.89 (95% CI ‐3.86 to 0.07) Relative % change 44.6% (95% CI 3.5 to 85.9) favoured exercise NNTB 2 (95% CI 1 to 34)	8 weeks: MD ‐0.68 (95% CI ‐1.62 to 0.26) (ns) 16 weeks: MD ‐1.79 (95% CI ‐2.70 to ‐0.88) (P < 0.001) 28 weeks: MD ‐0.85 (95% CI ‐1.77 to 0.07) (P = 0.07, ns) Overall (n = 180): MD ‐1.12 (95% CI ‐1.65 to ‐0.58) (P < 0.0001)	> 30% improvement post‐intervention.
Cramp 2013 (rheumatoid arthritis)	4 (not reported)	Not reported	In narrative only ‐ Harkcom 1985: statistics not reported separately for pain data, but reported as improvement over time; Hakkinen 2003: "stat significant improvement in 24 months"; Evans 2012 and Wang 2008: no statistically significant effects	Not reported	"Improvement over time" with "significant improvement in 24 months." No actual data available.
Fransen 2014 (OA)	End of treatment: 9 (549) 3 to 6 months: 5 (391)	Not reported; land based exercise vs no exercise: mean pain in control group ˜ 29/100 (based on 9 studies' control values)	End of treatment: SMD ‐0.38 (95% CI ‐0.55 to ‐0.20) "small to moderate" favoured exercise (P < 0.0001)	3 to 6 months: SMD ‐0.38 (95% CI ‐0.58 to ‐0.18) "small to moderate" favoured exercise (P = 0.0002)	"Small to moderate" statistically significant improvement, but only mild pain at baseline.
Fransen 2015 (OA)	End of treatment: 44 (3537) Follow‐up (2 to 6 months): 12 (1468) Follow‐up (> 6 months): 8 (1272)	Not reported; land‐based exercise vs no exercise: mean pain in control group 44/100 (based on 1 study control values)	Land‐based exercise vs no exercise: Mean pain in intervention groups was 0.49 SDs lower (95% CI 0.39 to 0.59 lower). This translates to an absolute mean reduction of 12 points (95% CI 10 to 15) compared with control group on a 0 to 100 scale. SMD ‐0.49 (95% CI ‐0.39 to ‐0.59) (P < 0.00001) Absolute reduction 12% (95% CI 10% to 15%) Relative change 27% (95% CI 21% to 32%) NNTB 4 (95% CI 3 to 5)	2 to 6 months: SMD ‐0.24 (95% CI ‐0.35 to ‐0.14) favoured exercise (P < 0.00001) > 6 months: SMD ‐0.52 (95% CI ‐1.01 to ‐0.03) favoured exercise (P = 0.04)	Absolute improvement of 12/100 post‐intervention (statistically significant).
Gross 2015a (mechanical neck disorders)	12‐week treatment: 2 (147) 24 week (or 12‐week treatment + 12‐week follow‐up): 2 (140)	Not reported, but control scores at end of treatment 40 to 60/100 (moderate pain)	12 weeks: pooled MD ‐14.90 (95% CI ‐22.40 to ‐7.39) favoured exercise (P = 0.0001)	24 weeks: pooled MD ‐10.94 (95% CI ‐18.81 to ‐3.08) favoured exercise (P = 0.0064)	2 trials showed a moderate (statistically significant) reduction in pain post‐intervention (14.9/100).
Hayden 2005 (low back pain)	Earliest follow‐up: 8 (370) Follow‐up (time since randomisation) Short term (6 weeks): 6 (268) Intermediate term (6 months): 5 (249) Long term (12 months): 2 (126)	"Chronic group" at baseline: mean 46/100 (95% CI 41 to 50) (moderate pain)	Earliest: MD ‐10.20 (95% CI ‐19.09 to ‐1.31) (P = 0.02)	Short term: MD ‐8.58 (95% CI ‐18.46 to 1.29) (P = 0.09, ns) Intermediate term: MD ‐12.48 (95% CI ‐22.69 to ‐2.27) (P = 0.02) Long term: MD ‐3.93 (95% CI ‐9.89 to 2.02) (P = 0.2, ns)	Reduction of ˜ 10/100 at earliest measurement point.
Hurkmans 2009 (rheumatoid arthritis)	4 studies (total 188 participants) in different categories (results not combined)	Not reported	Short‐term (12 weeks): Short‐term land‐based (aerobic and strength training) SMD ‐0.53 (95% CI ‐1.09 to 0.04) Short‐term land‐based (aerobic only) SMD ‐0.27 (95% CI ‐0.79 to 0.26) Short‐term water‐based SMD 0.06 (95% CI ‐0.43 to 0.54)	Long‐term (24 months) land‐based (aerobic and strength training) SMD 0.35 (95% CI ‐0.46 to 1.16)	No significant difference between control and intervention.
Koopman 2015 (postpolio syndrome)	1 (55)	Not reported, but control scores at end of treatment mean 44 (SD 24) on a 0 to 100 scale (moderate pain)	3 months post‐intervention: VAS (0 to 100): MD 11.00 (95% CI ‐0.98 to 22.98) (P = 0.072)	n/a	No significant effect/no difference between groups.
Regnaux 2015 (OA)	Only 1 study that had a no‐exercise control: 1 (68) ‐ excluded data for control (no exercise) from analysis (n = 34)	Not reported	Post‐intervention: WOMAC (0 to 20) Change data presented for high‐ vs low‐intensity groups only, not compared to control	n/a	Actual individual study data was extracted (where possible) instead of pooled MD or SMD due to comparison this overview wishes to make (exercise vs no‐exercise only). Could not extract exercise vs control data.
Saragiotto 2016 (low back pain)	Short term (< 3 months): 4 (291) Intermediate term (3 to 12 months): 4 (348) Long term (> 12 months): 3 (279)	Not reported, but control scores at follow‐up range 25 to 56/100 (mild‐moderate pain)	Short term: MD ‐10.01 (95% CI ‐15.67 to ‐4.35) favoured exercise (P < 0.001)	Intermediate term: MD ‐12.61 (95% CI ‐20.53 to ‐4.69) favoured exercise (P = 0.002) Long term: MD ‐12.97 (95% CI ‐18.51 to ‐7.42) favoured exercise (P < 0.001)	Medium effect size favouring exercise at all follow‐up assessments (moderate quality evidence at short‐ and long‐term, low quality evidence at intermediate term). Clinically important effect.
van der Heijden 2015 (patellofemoral pain syndrome)	3 studies with pain > 3 months (135 participants), 2 studies used in analysis (41 participants) Long‐term follow‐up: 1 (94)	Not reported, but control scores at follow‐up range 2.1 to 6.0/10 (mild‐moderate pain)	Short‐term (4 to 8 weeks): MD for usual pain in the exercise group was 0.93 (95% CI 1.60 to 0.25) SDs lower SMD ‐0.93 (95% CI ‐1.60 to ‐0.25) (P = 0.008)	"Long term" (16 weeks) VAS (0 to 10): MD ‐4.42 (95% CI ‐7.75 to ‐0.89) favoured exercise (P = 0.01)	Reduction in pain of 4/10 at 16 weeks' follow‐up.
Yamato 2015 (low back pain)	Short term: 6 (265) Intermediate term: 2 (148)	Not reported, but control scores at earliest follow‐up range 18 to 52/100 (mild‐moderate pain)	Short‐term follow‐up (< 3 months): MD ‐14.05 (95% CI ‐18.91 to ‐9.19) (P < 0.001)	Intermediate term (3 to 12 months): MD ‐10.54, (95% CI ‐18.54 to ‐2.62) (P = 0.009)	"Low quality evidence (downgraded due to imprecision and risk of bias) that Pilates reduces pain compared with minimal intervention at short‐term follow‐up, with a medium effect size... intermediate‐term follow‐up, two trials, provided moderate quality evidence (downgraded due to imprecision) that Pilates reduces pain compared with minimal intervention, with a medium effect size"

CI: confidence interval; HAQ: Health Assessment Questionnaire; MD: mean difference; n/a: not applicable; NNTB: number needed to treat for an additional beneficial outcome; ns: not significant; OA: osteoarthritis; SD: standard deviation; SF‐36: 36‐item Short Form; SMD: standardised mean difference; VAS: visual analogue score; WOMAC: Western Ontario and McMaster Universities Osteoarthritis Index; WUSPI; Wheelchair User Shoulder Pain Index.

**Table:** 

**Intervention group at baseline**	**Control group at baseline**	**Control group at earliest follow‐up**
Median pain score 70.9/100 (based on 7 studies, n = 382; Bidonde 2014)	WOMAC 9.1/20 (2 studies, n = 380) VAS ˜ 55/100 (3 studies, n = 117) HAQ 1.05/3 (1 study, n = 249) (Bartels 2007)	Mean pain score ˜ 29/100 (9 studies, n = 549; Fransen 2014)
11.05 to 22.6 on a 0 to 150 WUSPI score (1 study, n = 35; Boldt 2014)	VAS 35/100 to 61/100 (4 studies, n = 204; Busch 2007)	44/100 (44 studies, n = 3537; Fransen 2015)
Mean pain score 46/100 (95% CI 41 to 50) (8 studies, n = 370; Hayden 2005)	‐	40/100 to 60/100 (2 studies, n = 147; Gross 2015a)
‐	‐	44/100 SD 24 (1 study, n = 55; Koopman 2015)
‐	‐	range 25/100 to 56/100 (4 studies, n = 291; Saragiotto 2016)
‐	‐	2.1/10 to 6.0/10 (2 studies, n = 41; van der Heijden 2015)
‐	‐	range 18/100 to 52/100 (6 studies, n = 148; Yamato 2015)
**Range: 46 to 70.9 on a 0 to 100 scale** **16 studies, n = 787**	**Range: 35 to 55 on a 0 to 100 scale** **10 studies, n = 950**	**Range: 18 to 60 on a 0 to 100 scale** **68 studies, n = 4768**
CI: confidence interval; HAQ: Health Assessment Questionnaire; n: number of participants; SD: standard deviation; VAS: visual analogue score; WOMAC: Western Ontario and McMaster Universities Osteoarthritis Index; WUSPI: Wheelchair User's Shoulder Pain Index. HAQ: mean of different category scores, 0 or 1 (mild to moderate disability), up to 2 or 3 (severe to very severe disability); WOMAC pain score: 5 items summed to 0 (no pain) to 20 (worst pain ever); WUSPI: 15 items of 0 to 10 VAS scores, summed to form total of 0 (no pain) to 150 (worst pain ever).		

This suggests the majority of participants reviewed had mild‐to‐moderate pain (only one review reported a mean of severe pain (aquatic exercise for fibromyalgia, Bidonde 2014) at the commencement of each intervention (less than 30/100 mild pain, 30/100 to 60/100 moderate pain, more than 60/100 severe pain; Collins 1997), though labelling the majority as having only mild‐to‐moderate pain should be interpreted with caution due to the lack of specific data available ‐ the baseline data of the intervention group would have been preferable to the proxies we have had to use.

###### Quality judgement/ tiered quality (first, second, third tier evidence)

Our assessment criteria stated that we would accept the information as graded evidence when reported as the number of participants achieving a 50% (first tier evidence) or 30% (second tier evidence) reduction in pain, but none of the included reviews reported results in this way, and so instead we used the reported absolute and relative change values.

None of the included reviews fulfilled the requirements for first tier evidence (at least 50% pain reduction from baseline, study duration longer than eight weeks, and more than 200 participants per arm).

Second tier evidence (at least 30% pain reduction from baseline, study duration between four and eight weeks, and more than 200 participants in total or 100 participants per arm) was also lacking in these reviews; three reviews found at least 30% reduction in pain from baseline (Busch 2007; Busch 2013; van der Heijden 2015), one of which also used long enough exercise programmes (eight to 21 weeks' intervention, Busch 2013) but totalled only 81 participants across two studies. The other two reviews did not fulfil the study duration criteria (interventions from 2.5 weeks, Busch 2007; and three weeks, van der Heijden 2015) or study size criteria.

Consequently results from relevant reviews have been pooled (all tier three quality) where appropriate, though results should be interpreted with caution due to the low quality evidence.

###### Treatment effect

Data that could be extracted for pain can be seen in Table 9 for all reviews. Only three reviews found no statistically significant changes in usual or mean pain from any intervention (Cramp 2013; Hurkmans 2009; Koopman 2015 (assumed due to lack of presented data)). The remaining reviews reported a statistically significant effect of the intervention at one or more time points, in at least one subgroup.

Three reviews found at least 30% pain reduction from baseline (post‐intervention ‐ strength training: Busch 2007; Busch 2013, at short‐term follow‐up: van der Heijden 2015). Additionally, seven reviews reported clinically significant results (minimally important difference: reduction in pain from baseline of at least 10 points on a 0 to 100 scale or an absolute improvement of at least 10% to 20%, Dworkin 2008) as a result of the exercise intervention (1.3/10 from aerobic training, Busch 2007; 12/100 (95% CI 10 to 15), Fransen 2015,; 14.9/100 (95% CI 7.39 to 22.40), Gross 2015a; 10.2/100 (95% CI 1.31 to 19.09), Hayden 2005; 2.5/10 (95% CI 1.52 to 3.48), Boldt 2014; 10.01/100 (95% CI 4.35 to 15.67), Saragiotto 2016; 14.05/100 (95% CI 9.19 to 18.91), Yamato 2015). Three reviews found statistically significant improvements as a result of the intervention, but they did not reach clinical significance (post‐intervention, P = 0.02, Bartels 2007; "small to moderate" benefit post‐intervention and at six‐month follow‐up, P < 0.001, Fransen 2014; "moderate effect" of 7% (95% CI 3 to 11) benefit post‐intervention, Bidonde 2014).

Overall, results were inconsistent across interventions and follow‐up (see Table 9), as exercise did not consistently bring about a change (positive or negative) in self‐reported pain scores at any single point.

#### Secondary outcomes

##### Physical function (objectively or subjectively measured)

Measures of physical function were the primary outcome measure in eight out of 21 reviews (Busch 2013; Han 2004; Hayden 2005; Hurkmans 2009; Koopman 2015; Lane 2014; Lauret 2014; Silva 2010), and a reported (non‐primary) outcome measure in nine more reviews (Bartels 2007; Bidonde 2014; Busch 2007; Fransen 2014; Fransen 2015; Gross 2015a; Regnaux 2015; Saragiotto 2016; van der Heijden 2015, plus some which assessed disability; Cramp 2013; Saragiotto 2016; Yamato 2015). Only Boldt 2014 and Brown 2010 did not list physical function (or disability, or activity limitation) as a potential outcome measure.

###### Treatment effect

Data that could be extracted for physical function are shown in Table 10. Two reviews which reported physical function had no data to extract (Lauret 2014; Silva 2010), and for one review we were unable to extract the relevant data (Regnaux 2015). Two reviews found no significant difference in physical function between the intervention and control groups (Han 2004; Hurkmans 2009, both rheumatoid arthritis, 8 studies, n = 240). The remaining 14 reviews showed that the intervention produced a statistically significant benefit over the control at a minimum of one reported time point (Bartels 2007; Bidonde 2014; Busch 2007; Busch 2013; Cramp 2013; Fransen 2014; Fransen 2015; Gross 2015a; Hayden 2005; Koopman 2015; Lane 2014; Saragiotto 2016; van der Heijden 2015; Yamato 2015; 129 studies, n greater than 9559 (exact number unknown due to some participant numbers not being reported)).

**10:** Physical function

**Review**	**Outcome measure**	**Number of trials (and participants) used in analysis**	**Post‐intervention result (or if only 1 result reported)**	**Short‐term follow‐up (or if only 1 follow‐up point reported)**	**Intermediate‐term follow‐up**	**Long‐term follow‐up**	**Overall comment/statement**
Bartels 2007 (OA)	Self‐reported function (WOMAC and HAQ) and walking ability, and DRI	Post‐intervention Hip + knee function: 4 (648) walking ability: 2 (355) Hip only function: 1 (28) Follow‐up function hip + knee: 1 (306) hip only: 1 (17)	Function (hip + knee): SMD 0.26 (95% CI 0.11 to 0.42) favoured exercise (P < 0.001) Walking (hip + knee): SMD 0.18 (95% CI ‐0.03 to 0.39) favoured exercise (P = 0.08, ns) Function (hip only): SMD 0.76 (95% CI ‐0.02 to 1.53) favours exercise (P = 0.06, ns)	Hip only Disability, SMD 1.00 (95% CI ‐0.04 to 2.04) favoured exercise (P = 0.06, ns)	Hip + knee (6 months) Function, SMD 0.10 (95% CI ‐0.12 to 0.33) (ns)	n/a	Function was significantly improved in people with hip + knee OA immediately post‐intervention only ‐ small effect size only.
Bidonde 2014 (fibromyalgia)	Self‐reported physical function (0 to 100 scale)	5 (285)	MD ‐4.35 (95% CI ‐7.77 to ‐0.94) SMD ‐0.44 (95% CI ‐0.76 to ‐0.11) Absolute difference ‐4 (95% CI ‐8 to ‐1) NNTB 6 (95% CI 3 to 22)	n/a	n/a	n/a	Small difference (improvement) in aquatic exercise group. Among the major wellness outcomes, none of the outcomes met the threshold for clinically relevant differences (15%).
Busch 2007 (fibromyalgia)	Physical function	Aerobic: 4 (253) Strength: 2 (47)	Aerobic: SMD 0.66 (95% CI 0.41 to 0.92) favoured exercise (P < 0.0001) Strength: SMD 0.52 (95% CI ‐0.07 to 1.10) favoured exercise (P = 0.08, ns)	n/a	n/a	n/a	Function was significantly improved from aerobic exercise training, strength training neared significance. Moderate effect size.
Busch 2013 (fibromyalgia)	HAQ and SF‐36 for function	3 (107)	Change score MD ‐6.29 (95% CI ‐10.45 to ‐2.13) favoured exercise (P < 0.01)	n/a	n/a	n/a	Significantly favourable effect of exercise.
Cramp 2013 (rheumatoid arthritis)	Disability	4 (not reported)	n/a	n/a	n/a	n/a	"Studies investigating hydrotherapy and tai chi demonstrated statistically significant improvements in the intervention arm compared to the control arm between baseline and follow‐up. The studies investigating strength training and Ivengar yoga did not demonstrate a statistically significant difference between study arms."
Fransen 2014 (OA)	Physical function	Post‐intervention: 9 (521) Follow‐up (3 to 6 months): 5 (365)	SMD ‐0.30 (95% CI ‐0.54 to ‐0.05) "significant benefit" favoured exercise (P = 0.02) The demonstrated effect size for exercise was equivalent to an improvement of physical function of 7 points (95% CI 1 to 12) on a 0 to 100 scale compared with a control group	SMD ‐0.37 (95% CI ‐0.57 to ‐0.16) favoured exercise (P < 0.001)	n/a	n/a	Statistically significant, but small effect size only.
Fransen 2015 (OA)	Physical function	Post‐intervention: 44 (3913) Follow‐up (2 to 6 months): 10 (1279) Follow‐up (> 6 months): 8 (1266)	SMD ‐0.52 (95% CI ‐0.64 to ‐0.39) favoured exercise (P < 0.0001); an improvement of 10 points (95% CI 8 to 13) on a 0‐ to 100‐point scale	SMD ‐0.15 (95% CI ‐0.26 to ‐0.04) favoured exercise (P = 0.008)	SMD ‐0.57 (95% CI ‐1.05 to ‐0.10) favoured exercise (P = 0.02)	n/a	Significant effect from exercise at every follow‐up point. Moderate effect size at short‐ and long‐term follow‐up, but only small effect at intermediate‐term follow‐up.
Gross 2015a (mechanical neck disorders)	Physical function	12 wk: 2 (147) 24 wk: 2 (140)	12 wk treatment: pooled SMD ‐0.50 (95% CI ‐1.04 to 0.03) favoured exercise (P = 0.07, ns)	24 wk treatment (or 12 wk' treatment + 12 wk follow‐up): pooled SMD ‐0.40 (95% CI ‐0.74 to ‐0.06) favoured exercise (P = 0.02)	n/a	n/a	2 trials showed a moderate (statistical) improvement in function.
Han 2004 (rheumatoid arthritis)	Functional assessment and 50‐feet walk test	Function: 2 (52) Walk test: 2 (48)	Function: MD 0.01 (95% CI ‐2.94 to 2.97) (ns) Walk test: MD 0.35 seconds (95% CI ‐1.14 to 1.84) (ns)	n/a	n/a	n/a	No significant effect.
Hayden 2005 (low back pain)	Function	Earliest: 7 (337) Short term: 6 (268) Intermediate term: 4 (216) Long term: 2 (126)	Earliest: MD ‐2.98 (95% CI ‐6.48 to 0.53) favoured exercise (P = 0.09, ns)	Short term: MD ‐3.03 (95% CI ‐6.35 to 0.53) favoured exercise (P = 0.07, ns)	Intermediate term: MD ‐3.84 (95% CI ‐7.06 to ‐0.61) favoured exercise (P = 0.02)	Long term: MD ‐4.22 (95% CI ‐7.99 to ‐0.46) favoured exercise (P = 0.03)	Favoured exercise from the earliest measure, but only reached statistical significance at intermediate and long term after randomisation.
Hurkmans 2009 (rheumatoid arthritis)	Functional ability	Land‐based aerobic: 2 (66) Land‐based aerobic + strength: 2 (74)	n/a	Short‐term training (12 wk) Land‐based aerobic only training SMD 0.03 (95% CI ‐0.46 to 0.51) (ns) Land‐based aerobic and strength training SMD ‐0.4 (95% CI ‐0.86 to 0.06) (ns)	n/a	n/a	No significant difference between control and intervention groups.
Koopman 2015 (postpolio syndrome)	Muscle strength; and activity limitation (Sunnaas ADL‐index range 0 to 36; Rivermead Mobility Index (RMI) range 0 to 15)	Strength: 1 (10) Activity limitation: 1 (53)	Isometric muscle strength (postintervention): MD 39.00% (95% CI 6.12 to 71.88) Activity limitation: 3 months' postintervention: ADL‐index: MD ‐2.70 (95% CI ‐4.53 to ‐0.87) Rivermead Mobility Index (RMI): MD ‐1.50 (95% CI ‐2.93 to ‐0.07)	Activity limitation: 6‐months post‐intervention: ADL‐index: MD ‐2.90 (95% CI ‐4.73 to ‐1.07) RMI: MD ‐1.80 (95% CI ‐3.19 to ‐0.41)	n/a	n/a	Activity limitation: favoured intervention at both assessment points. "The baseline imbalance in favour of the usual care group probably biased these results."
Lane 2014 (intermittent claudication)	Maximal walking time and maximal walking distance	Post‐intervention Walking time: 12 (577) Walking distance: 9 (480) 3‐month follow‐up Walking time: 5 (174) Walking distance: 3 (116) 6‐month follow‐up Walking time: 4 (295) Walking distance: 3 (156)	Time: MD 4.51 minutes (95% CI 3.11 to 5.92) favoured exercise (P < 0.00001) Distance: 108.99 m (95% CI 38.20 to 179.78) favoured exercise (P = 0.003)	Time: MD 6.05 minutes (95% CI 5.47 to 6.62) favoured exercise (P < 0.00001) Distance: MD 104.46 m (95% CI ‐64.33 to 273.24) favoured exercise (ns)	Time: MD 3.20 minutes (2.04 to 4.36) favoured exercise (P < 0.0001) Distance: MD 138.36 m (95% CI 22.39 to 254.34) favoured exercise (P = 0.02)	n/a	Objectively measured walking time and distance showed significant improvement.
Lauret 2014 (intermittent claudication)	Maximal walking time (mins) and maximal walking distance (metres)	No relevant studies	n/a	n/a	n/a	n/a	No relevant studies.
Regnaux 2015 (OA)	WOMAC (0 to 68) disability scale, and muscle strength	1 (68) ‐ excluded control (no‐exercise data: n = 34)	n/a	n/a	n/a	n/a	Could not extract exercise vs control data ‐ data presented for high vs low intensity groups only, not compared to control.
Saragiotto 2016 (low back pain)	Disability (Oswestry Disability Index, Roland Morris Disability Questionnaire)	Short‐term follow‐up (< 3 months): 5 (332) Intermediate term (3 to 12 months): 4 (348) Long term (> 12 months): 3 (279)	‐	MD ‐8.63 (95% CI ‐14.78 to ‐2.47) (P < 0.01)	MD ‐5.47 (95% CI ‐9.17 to ‐1.77) (P = 0.004)	MD ‐5.96 (95% CI ‐9.81 to ‐2.11) (P = 0.002)	Small effect sizes, favoured exercise. Short term: CI included a clinically important effect.
Silva 2010 (rheumatoid arthritis)	HAQ function	No studies found	n/a	n/a	n/a	n/a	No studies found.
van der Heijden 2015 (patellofemoral pain syndrome)	Functional ability	Short‐term follow‐up: 7 (483) Long‐term follow‐up: 3 (274)	n/a	Short‐term (4 to 8 wk): SMD 1.10 (95% CI 0.58 to 1.63) favoured exercise (P < 0.0001)	n/a	SMD 1.62 (95% CI 0.31 to 2.94) favoured exercise (P = 0.02)	Significant effect of exercise. Very large effect size at short‐ and long‐term follow‐up.
Yamato 2015 (low back pain)	Disability (all measures converted to 0 to 100 scale)	Short‐term (< 3 months) follow‐up: 5 (248) ‐Intermediate‐term (3 to 12 months) follow‐up: 2 (146)	n/a	MD ‐7.95 (95% CI ‐13.23 to ‐2.67) (P = 0.003)	MD ‐11.17 (95% CI ‐18.41 to ‐3.92) (P = 0.0025)	n/a	"Low quality evidence (downgraded due to imprecision and inconsistency) that Pilates improves disability at short‐term follow‐up compared with minimal intervention, with a small effect size .... intermediate‐term follow‐up, two trials provided moderate quality evidence (downgraded due to imprecision) of a significant effect in favour of Pilates, with a medium effect size"

ADL: activities of daily living; CI: confidence interval; DRI: Disability Rating Index; HAQ: Health Assessment Questionnaire; MD: mean difference; n/a: not applicable; NNTB: number needed to treat for an additional beneficial outcome; ns: not significant; OA: osteoarthritis; SF‐36: 36‐item Short Form; SMD: standardised mean difference; wk: week; WOMAC: Western Ontario and McMaster Universities Osteoarthritis Index,

Many of these statistically significant results were of small or moderate effect size (as reported by the review authors, or using the definition by Cohen 1988 if unreported; small effect size: Bartels 2007; Bidonde 2014; Fransen 2014; Fransen 2015; Gross 2015a; Koopman 2015; Saragiotto 2016; Yamato 2015, moderate effect size: Busch 2007; Fransen 2015; Yamato 2015).

Only one review reported statistical significance and large effect size (both short‐term and long‐term follow‐up: SMD 1.10 (95% CI 0.58 to 1.63) and 1.62 (95% CI 0.31 to 2.94), van der Heijden 2015). However, the original review authors highlighted the low to very low quality of the evidence as many studies had high or unclear risk of bias across multiple domains (van der Heijden 2015).

##### Psychological function

Only five out of 21 reviews assessed psychological function as mental health (Bartels 2007; Bidonde 2014; Busch 2013), anxiety (Cramp 2013), and depression (Boldt 2014; Busch 2013; Cramp 2013).

###### Treatment effect

Data that could be extracted for psychological function can be seen in Table 11. There were significant effects in favour of the intervention for mental health (Bartels 2007) and depression (Busch 2013) scores, and "variable effect" for depression (Cramp 2013). However, there was also no effect or no differences between control and intervention groups reported for mental health (Bidonde 2014; Busch 2013), anxiety (Cramp 2013), and depression (Boldt 2014).

**11:** Psychological function

**Review**	**Outcome measure**	**Number of trials (and participants) reporting psychological function**	**Outcome result (postintervention or if only one measurement point)**	**Follow‐up**	**Additional statement/comment**
**Mental health**					
Bartels 2007	‐	4 studies	SMD 0.16 (95% CI 0.01 to 0.032) favoured aquatic exercise	No significant difference at 6 months, 1 study	Very small effect size postintervention.
Busch 2013	SF‐36 ‐ Mental health scale	1 study	‐	n/a	No group differences.
Bidonde 2014	SF‐36 ‐ mental Health scale SF‐12 ‐ Mental Health scale	4 studies, n = 243	MD ‐3.03 (95% CI ‐8.06 to 2.01)	n/a	No effect.
**Anxiety**					
Cramp 2013	Brief Symptom Inventory	1 study	"No significant effect"	n/a	‐
**Depression**					
Boldt 2014	CES‐D	1 study, n = 34	MD ‐6.0 (95% CI ‐15.87 to 3.87) (P = 0.23)	n/a	No effect.
Busch 2013	HADS ‐ Depression Beck Depression Index	1 study, n = 21	MD ‐3.70 (95% CI ‐6.37 to ‐1.03) Relative difference 57%	n/a	Significant effect, favoured resistance training.
Cramp 2013	CES‐D	Not reported	"Variable effect" reported in text only	n/a	‐

CES‐D: Centre for Epidemiological Studies‐Depression; CI: confidence interval; HADS: Hospital Anxiety and Depression Scale; MD: mean difference; n: number of participants; n/a: not applicable; SF‐12: 12‐item Short Form; SF‐36: 36‐item Short Form; SMD: standardised mean difference.

##### Quality of life

A version of quality of life assessment was reported in nine reviews. Six were termed quality of life or health‐related quality of life (HRQoL) (Bartels 2007; Boldt 2014; Fransen 2014; Fransen 2015; Gross 2015a; Lauret 2014).

Other reviews assessed global perceived effect (Gross 2015a), global wellbeing (Busch 2007), global assessment (Hayden 2005), global impression of recovery (Saragiotto 2016; Yamato 2015), health assessment questionnaire (Silva 2010), multi‐dimensional function (Bidonde 2014; Busch 2013), and work status (Hayden 2005). These have been reported separately to quality of life (Table 12).

**12:** Quality of life

**Review**	**Outcome measure**	**Number of trials (and participants) reporting Quality of Life (QoL)**	**Outcome result**	**Additional statement/comment**
**(Health‐related) Quality of Life**				
Bartels 2007	QoL: SF‐12 (Physical), PQoL, EuroQoL	Hip + knee OA (post‐intervention): 3 studies, n = 599 Hip only OA (post‐intervention): 1 study, n = 28 Hip only OA (follow‐up): 1 study, n = 17	Hip + knee (post‐intervention): SMD 0.32 (95% CI 0.03 to 0.61) (P = 0.028) Hip only (post‐intervention): SMD 0.76 (95% CI ‐0.02 to 1.53) (ns) Hip only (follow‐up): SMD 1.00 (95% CI ‐0.04 to 2.04) (ns)	Significantly favoured aquatic exercise post‐intervention in hip + knee OA. Small effect size only (when statistically significant).
Boldt 2014	PQoL (perceived quality of life) SQoL (subjective quality of life)	Post‐intervention: 1 study, n = 34, PQoL; 1 study, n = 80, SQoL Follow‐up (intermediate term): 1 study, n = 80, SQoL	Post‐intervention: PQoL MD 10.8 (95% CI ‐4.2 to 25.8) (P = 0.16) SQoL MD 0.3 (95% CI ‐0.22 to 0.82) (P = 0.25) Follow‐up: SQoL MD 0.5 (95% CI ‐0.03 to 1.03) (P = 0.07)	No difference between groups.
Fransen 2014	QoL	Post‐intervention: 3 studies, n = 183	SMD 0.07 (95% CI ‐0.23 to 0.36) (ns)	No difference between groups.
Fransen 2015	QoL: self‐report questionnaire, scale 0 to 100 (100 is maximum QoL)	Post‐intervention: 13 studies, n = 1073	SMD 0.28 (95% CI 0.15 to 0.40) (P < 0.0001) Absolute difference 4% (95% CI 2% to 5%) relative difference 9% (95% CI 5% to 13%)	Statistically significant, but equates to an absolute improvement of 4 points (95% CI 2 to 5) on a 0 to 100 scale. Small effect size only.
Gross 2015a	QoL: SF‐36 (Physical Function subscale)	Post‐intervention: 2 studies, n = 143	12‐wk intervention: MD ‐2.22 (95% CI ‐5.17 to 0.72) (ns) 24‐wk intervention: MD 0.06 (95% CI ‐4.06 to 4.17) (ns)	No significant difference between groups.
Lauret 2014	HRQoL	No relevant studies	n/a	n/a
**Global assessment**				
Busch 2007	Global wellbeing	Strength: 2 studies, n = 47 Aerobic: 4 studies, n = 269	Strength: SMD 1.43 (95% CI 0.76 to 2.10) Aerobic: SMD 0.49 (95% CI 0.23 to 0.75)	Favoured exercise ‐ higher score showed better QoL, Strength: very large effect size. Aerobic: small‐to‐moderate effect size only.
Bidonde 2014	Participant‐rated global (10‐cm VAS)	1 study, n = 46	MD ‐0.87 (95% CI ‐1.74 to 0.00)	No effect.
Gross 2015a	Global perceived effect	1 study, n = 70	"No significant difference"	No significant difference.
Hayden 2005	Global assessment	7 studies, n = 16	Not reported	n/a
Saragiotto 2016	Global impression of recovery	1 study, n = 154	Short term, MD 1.30 (95% CI 0.30 to 2.30) (P = 0.01) Intermediate term, MD 1.20 (95% CI 0.31 to 2.09) (P = 0.008) Long term, MD 1.50 (95% CI 0.61 to 2.39) (P < 0.001)	Medium effect size.
Yamato 2015	Global impression of recovery	1 study, n = 86	Short term (< 3 months): MD 1.50 (95% CI 0.70 to 2.30) Intermediate term (3 to 12 months): MD 0.70 (95% CI ‐0.11 to 1.51)	"Low quality evidence (downgraded due to imprecision and inconsistency), we found a significant short‐term effect, with a small effect size, but not for intermediate/mid‐term follow up."
**Other method of assessment**				
Bidonde 2014	Multi‐dimensional function‐ FIQ	7 studies, n = 367	MD ‐5.97 (95% CI ‐9.06 to ‐2.88) SMD ‐0.55 (95% CI ‐0.83 to ‐0.27) Absolute difference ‐6 (95% CI ‐9 to ‐3) NNTB 5 (95% CI 3 to 9)	Favoured aquatic exercise ‐ lower score showed reduced impact of pain on life. "Moderate difference."
Busch 2013	Multi‐dimensional function ‐ FIQ	1 study, n = 60	SMD ‐1.27 (95% CI ‐1.83 to ‐0.72) Absolute difference ‐16.75 FIQ units (95% CI ‐23.31 to ‐10.19)	Favoured exercise ‐ lower score showed reduced impact of pain on life. Very large effect size.
Hayden 2005	Work status	9 studies, n = 21	Not reported	n/a
Silva 2010	Health Assessment Questionnaire (HAQ)	No included studies	n/a	n/a

FIQ: Fibromyalgia Impact Questionnaire; HRQoL: health‐related quality of life; MD: mean difference; n: number of participants; n/a: not applicable; NNTB: number needed to treat for an additional beneficial outcome; OA: osteoarthritis; PQoL: perceived quality of life; QoL: quality of life; SF‐36: 36‐item Short Form; SMD: standardised mean difference; SQoL: subjective quality of life; VAS: visual analogue scale.

###### Treatment effect

Data that could be extracted for quality of life can be seen in Table 12. Four reviews found no significant difference between intervention and control groups in health‐related quality of life post‐intervention (9 studies, n = 556) (HRQoL: Boldt 2014; Fransen 2014; Gross 2015a, global assessment: Bidonde 2014; Gross 2015a)), three reviews did not or were unable to report any data (HRQoL: Lauret 2014, global assessment: Hayden 2005, other assessment: Silva 2010), and seven reviews found a significant improvement as a result of the intervention (34 studies, n = 2700) (HRQoL: Bartels 2007, Fransen 2015, global assessment: Busch 2007; Saragiotto 2016; Yamato 2015, other assessment: Bidonde 2014; Busch 2013).

Two reviews assessing strength/resistance training interventions found significantly large effect sizes (SMD greater than 0.8, as defined by Cohen 1988) in favour of the intervention (global wellbeing measure, SMD 1.43 (95% CI 0.76 to 2.10), Busch 2007; Fibromyalgia Impact Questionnaire, SMD 1.27 (95% CI 0.72 to 1.83), Busch 2013). Other statistically significant changes reported in the included reviews were of small‐to‐moderate effect size (SMD 0.2 to 0.8, Cohen 1988).

##### Adherence to the prescribed intervention

Only one review reported adherence to the intervention as an outcome measure (Regnaux 2015), but the authors were unable to perform an analysis on attendance as most studies did not clearly report attendance or compliance (Regnaux 2015). However, five reviews assessed withdrawals or dropouts (Bidonde 2014; Fransen 2014; Han 2004; Regnaux 2015; Saragiotto 2016), one reported all‐cause attrition (Busch 2013), and another reported the discontinuation rate (Silva 2010).

Data that could be extracted for adherence, withdrawals, and attrition can be seen in Table 13. Pooling all available data for withdrawals/dropout/attrition gave an RR of 1.02 (95% CI 0.94 to 1.12) in favour of the control group (6 reviews, 30 studies, n = 2256, control withdrawal 81/1000, intervention withdrawal 82.8/1000).

**13:** Adherence/withdrawals

**Review**	**Number of trials (and participants) reporting withdrawals**	**Number withdrawn (per 1000) ‐ intervention group**	**Number withdrawn (per 1000) ‐ control group**	**RR or OR**
Bidonde 2014 (fibromyalgia)	8 studies, n = 472	151 (imputed from reported 38/252)	129 (imputed from reported 30/232)	RR 1.13 (95% CI 0.73 to 1.77) (P = 0.45)
Busch 2013 (fibromyalgia)	3 studies, n = 107	134 (95% CI 30 to 439)	39	RR 3.50 (95% CI 0.79 to 15.49)
Fransen 2014 (osteoarthritis)	7 studies, n = 715	59 (95% CI 30 to 114)	34	OR 1.77 (95% CI 0.86 to 3.65)
Han 2004 (rheumatoid arthritis)	4 studies, n = 189	109 (imputed from reported 11/101)	284 (imputed from reported 25/88)	RR 0.37 (95% CI 0.19 to 0.72)
Regnaux 2015 (osteoarthritis)	1 study, n = 102	44 (imputed from reported 3/68 (4%); all from high‐intensity group)	0	Calculated RR 3.55 (95% CI 0.19 to 66.8)
Saragiotto 2016 (low back pain)	7 studies, n = 671	0	0	‐
Silva 2010 (rheumatoid arthritis)	No included studies	n/a	n/a	n/a
**Total**	**30 studies, n = 2256**	**82.8/1000**	**81/1000**	**Calculated RR 1.02 (95% CI 0.94 to 1.12)** **Calculated OR 1.05 (95% CI 0.88 to 1.25)**

CI: confidence interval; n: number of participants; n/a: not applicable; OR: odds ratio; RR: risk ratio.

One clinically controlled trial (CCT) in one review reported statistically significant improvement in enjoyment of exercise/rest (P = 0.0002) and self‐reported benefit from exercise/rest (P = 0.006) at both post‐intervention (end of therapy, 10 weeks) and follow‐up (four months later) (n = 95, Han 2004).

##### Healthcare use/attendance

None of the reviews reported healthcare use/attendance.

##### Adverse events (not death)

Eighteen out of 21 reviews reported adverse effects (three reviews did not report adverse events as an outcome measure due to lack of studies or other undisclosed reasons; Brown 2010; Lauret 2014; Silva 2010). Two reviews only assessed a specific adverse event ("amputation" Lane 2014; "motor unit survival" Koopman 2015), one review observed "safety ‐ pain and radiological damage" (Hurkmans 2009), and another referred to any "side‐effects" (Han 2004).

Data that could be extracted for adverse events (not death) can be seen in Table 14. The total number of reported adverse events (not death) was 137 events across 39 studies out of 61 studies that had adverse events as an outcome measure (over one‐third of all trials that reported them found no adverse events related to the intervention): six reviews reported no adverse events from the included trials (Bartels 2007; Busch 2013; Cramp 2013; Hurkmans 2009; Koopman 2015; Yamato 2015) though the authors questioned whether this was due to lack of reporting by the trial authors, or whether there were no adverse events.

**14:** Adverse events (not death)

**Review**	**Total number of trials (and participants) in review reporting exercise vs control in chronic pain population**	**Number of trials (and participants) reporting adverse events**	**Number of adverse events**	**Overall statement**
Bartels 2007	4 (674)	2 (148)	0	Adverse events were recorded (and reported), but none occurred.
Bidonde 2014	9 (519)	0	0	Review stated that no included studies actively reported on adverse events (some reported withdrawal).
Boldt 2014	3 (149)	2 (115)	5 events over 2 studies	"Neck, shoulder and elbow injuries in five participants in the intervention group."
Busch 2007	34 (2276)	6 (strength training: 115, aerobic: 1264)	Strength training: 3 Aerobic training: 5	‐
Busch 2013	3 (81)	2 (86 exercising participants)	0	Adverse events were recorded (and reported), but none occurred.
Cramp 2013	6 (371)	3	0	Adverse events were recorded (and reported), but none occurred.
Fransen 2014	10 (> 549)	5	7 events over 3 studies	‐
Fransen 2015	54 (5362)	11	42 events over 8 studies	‐
Gross 2015a	16 (2485)	11	41 events over 6 studies	‐
Han 2004	3 (206)	2	1 event in 1 study	In narrative: "approximately one‐third of the patients complained of soreness in the knee, shoulder or lower back during the first 3 weeks… pain eventually subsided for all patients… only exception was one patient, who complained of knee pain."
Hayden 2005	43 (3907)	10	23 events over 10 studies	"Negative reported: 16 events over 7 trials."
Hurkmans 2009	5 (575)	2	0	Adverse events were recorded (and reported), but none occurred.
Koopman 2015	2 (68)	1 (10)	0	Adverse events were recorded (and reported), but none occurred. "The study investigated deleterious effects of this training on motor unit survival through motor unit number estimates (MUNE). Results showed that the MUNE did not change at the end of the training."
Lane 2014	30 (1822)	1 (88 exercising participants)	2 events in control group in 1 study	RR 0.20 (95% CI 0.01 to 4.15) in favour of exercise group.
Regnaux 2015	1 (102)	1 (68 exercising participants over 2 groups: low and high resistance)	3 events in 1 study	"3 participants in high resistance group discontinued the exercise intervention due to severe knee pain."
Saragiotto 2016	7 (671)	1 (154)	5 events in 1 study	"Five patients (three from the MCE [motor control exercise] group and two from the minimal intervention group) had mild adverse effects during the study (all temporary exacerbations of pain)."
van der Heijden 2015	10 (1690)	0	0	Of the relevant studies, none actively reported on adverse events.
Yamato 2015	6 (265)	1 (86)	0	Adverse events were recorded (and reported), but none occurred.
**Total**	**246 studies ** **(> 21,772)**	**61 studies ** **(> 2134 participants)**	**137 events over 39 studies**	**61/246 (25%) of studies have reported on adverse events; of which 39/61 (64%) did have adverse events occur as a result of the intervention or control.**

n: number of participants; RR: risk ratio.

Adverse events were largely reported as a total number per trial, though one review separately reported results for the intervention group versus the control group (Saragiotto 2016), and two others reported adverse events for the intervention group only (Boldt 2014; Regnaux 2015). Only one review calculated an RR for the adverse events, showing a reduced risk for amputation in the intervention group (two amputations in the usual care/control group: RR 0.20, 95% CI 0.01 to 4.15, based on one study in one review, Lane 2014).

##### Death

Only one out of 21 reviews reported death separately to other adverse events (Lane 2014). Based on five studies within the review, death had an RR of 0.71 (95% CI 0.28 to 1.78) in favour of exercise as being protective, though was not statistically significant (P = 0.47).

## Discussion

**Specificity of the condition:** despite the heterogeneous nature of chronic pain, in this overview we have combined several painful conditions covering a number of conditions and diagnoses. Regardless of aetiology, the impact of chronic pain is broadly similar across many conditions.

### Summary of main results

**Pain severity:** there were favourable results in a number of reviews as a result of exercise: only three reviews found no statistically significant changes in usual or mean pain from any intervention. However, results were inconsistent across interventions and follow‐up, as the intervention did not consistently bring about a change (positive or negative) in self‐reported pain scores at any single point. The exercise or physical activity interventions did not have a negative effect on the outcome (did not worsen the pain). A factor in the lack of statistical and clinically significant result may be the baseline pain severity of participants. The majority of the included population had an assumed mild‐to‐moderate pain severity score (assumed only due to lack of exact group data at baseline). This is often the desired outcome (post‐intervention) of many drug therapies for pain, and it may therefore be difficult to show a clinically significant improvement in these people.

**Physical function:** physical function/disability was the most commonly reported outcome measure, and was the primary measure in eight out of the 21 reviews. Physical function was significantly (statistically) improved as a result of the intervention in 14 reviews, though even these statistically significant results had only small‐to‐moderate effect sizes in all but one review.

**Psychological function and quality of life:** there were variable results for psychological function and quality of life: results were either favourable to exercise (two reviews reporting significantly large effect sizes for quality of life), or showed no difference between groups. There were no negative effects.

**Adherence to the prescribed intervention:** could not be assessed in any included review. However, risk of withdrawal/dropout was slightly higher in the exercising group (82.8/1000 participants versus 81/1000 participants), though the group difference was not significant.

**Healthcare use/attendance:** not reported in any included review.

**Adverse events, potential harm, and death:** importantly, exercise caused no actual harm, with most adverse events being increased soreness or muscle pain, which reportedly subsided after several weeks of the intervention. One review reported a non‐significant reduction in risk of death as a result of the intervention.

### Overall completeness and applicability of evidence

Of the 21 included reviews, seven could be considered out of date as they were most recently assessed as up‐to‐date prior to 2010 such that any recent controlled trials assessing pain severity have not been included in this overview (Cochrane recommends updating reviews every two years) (Bartels 2007; Brown 2010; Busch 2007; Han 2004; Hayden 2005; Hurkmans 2009; Silva 2010). We included these reviews in the overview, but they may not be as relevant now due to the elapsed time since they were updated. One protocol that had potential to be included was published in 2006 with no full review available yet (Craane 2006).

Available data suggest that participants in the included reviews and studies would generally be characterised as having mild‐moderate pain (moderate greater than 30/100 or 3/10) with only one review reporting moderate‐severe pain (severe greater than 60/100 or 6/10). Therefore whether the evidence of change or no change seen here as a result of each intervention is applicable to people further along on the pain spectrum (with higher pain scores/worse pain) is debatable. However, it can be argued that those people are more likely to be assigned medical or surgical interventions than physical activity and exercise alone (where available), and as a group they may be less able to engage in exercise, and may therefore be more difficult to recruit into exercise‐only studies. Having said this, the labelling of participants as having mild‐moderate pain was a cautious one within this overview due to the lack of specific data available at baseline assessment; only three reviews included baseline pain scores in the intervention group, and two further reviews provided control group baseline scores.

There are still gaps in the available literature, and therefore also within this overview. None of the included reviews examined generalised or widespread chronic pain as a global condition, each instead examined specific conditions that included chronic pain as a symptom or result of the ongoing condition (rheumatoid arthritis, osteoarthritis, fibromyalgia, low back pain, intermittent claudication, dysmenorrhoea, mechanical neck disorder, spinal cord injury, postpolio syndrome, and patellofemoral pain). The pain in these cases can occur secondary to other symptoms such as fatigue, muscle stiffness, difficulty sleeping, and depression, all of which could separately (and more effectively) be influenced by the intervention. Additionally, only 25% of included studies actively reported adverse events. This may affect the completeness of the evidence as conclusions have been drawn based on the available data. The included reviews did not discuss the possible impact of this non‐reporting by the original trials, and this may lead to underestimating possible adverse events from an intervention, or overestimating its safety.

The exercise interventions examined in the included reviews were broad; including aerobic, strength, flexibility, range of motion, and core or balance training programmes, as well as yoga, Pilates, and tai chi. Many of these interventions can be accessed in the community by the general public and people with chronic pain, either individually or in classes (yoga, Pilates, tai chi). Other exercise intervention programmes, such as the motor control exercise and proprioceptive (balance) training, requires at least initial supervision by a therapist to teach the correct techniques and provide feedback for progression.

### Quality of the evidence

In assessing the quality of the evidence, we employed the AMSTAR tool to examine the reviews, extracted data on risk of bias to examine the available primary evidence, and evaluated the authors' conclusions to ensure that they were appropriate based on the available data.

The AMSTAR tool is useful in assessing the reporting of a systematic review, though it does not inform us of the actual undertaking or conduct of the review process. All 21 included reviews scored well across the AMSTAR assessment, though this is likely due to the stringent reporting guidelines implemented by Cochrane prior to publication. However, it may be necessary or advisable for the Cochrane guidelines to be further expanded and detailed with regards to reporting study characteristics, publication bias, and conflicts of interest, as these areas often did not meet the requirements laid out in the AMSTAR criteria (Table 1).

Data extracted from the reviews regarding their assessment of bias (risk of bias) showed moderate level scores at best across all included studies within the included reviews. Other than issues surrounding blinding (which are problematic in exercise intervention studies due to the nature of the intervention), the trials did not consistently and adequately report potential attrition and reporting biases, with less than half of studies within these reviews at low risk of bias.

However, the most prominent issue with regards to bias in these exercise and physical activity intervention studies is the sample size used. This subcategory is not used as standard in the assessment of bias in Cochrane Reviews, despite the increasing volume of research available suggesting that small studies of fewer than 100 participants per arm (Moore 2010; Nüesch 2010) are at increased risk of succumbing to the random effects in estimating both direction and magnitude of treatment effects (Moore 1998; Turner 2013) due to greater heterogeneity within and between small studies (IntHout 2015).

Studies within the included reviews here were very small (often fewer than 50 participants in total). For greater quality and a more reliable effect, at least 100 participants per arm should be analysed for a study to potentially be classed as tier two evidence (200 per arm for tier one); small studies are known to overestimate the treatment effect by up to 32% in comparison with larger studies (Deschartes 2013).

Assessing studies for risk of bias based on study size (total number or per arm) should be included in any review or meta‐analysis in future, to adequately assess the influence of small trials on the estimated treatment effect (Nüesch 2010). Inclusion in the standard assessment process may in turn influence the design and undertaking of future research trials to increase the sample size, and produce more consistent clinically and statistically accurate results.

Of the 21 included reviews, 12 used a pain measure as their primary outcome (Bartels 2007; Boldt 2014; Brown 2010; Busch 2007; Fransen 2014; Fransen 2015; Gross 2015a; Hayden 2005; Regnaux 2015; Saragiotto 2016; van der Heijden 2015; Yamato 2015), and the remaining nine reviews included the measure as a secondary outcome only. Other outcomes were shared, including physical and psychological function, and quality of life. Likewise, each review team will have included studies that did not use their chosen outcome measures as the primary measure, and that were therefore powered according to a different primary outcome. On collating the evidence, some studies may appear underpowered for the outcome(s) of interest to us (Turner 2013), yet were adequately powered for the studies' primary measure. To increase the power of the results of this overview, and the intermediary reviews we have included, intervention studies that focus on painful conditions should include pain intensity as the primary outcome, or at least as a prominent secondary outcome; alternatively review authors should seek to include only those studies that were adequately powered for pain intensity as a primary outcome measure.

Intervention length ranged from a single session to regular sessions over a period of 30 months, though the majority were between eight and 12 weeks. Durations of this length are common among exercise and physical activity intervention studies to allow for physiological adaptation and familiarisation. In contrast, the follow‐up period was often inadequate, as many reviews reported only a single follow‐up point (immediately post‐intervention), or repeated measures over the short‐term (less than six months): only six of the 21 reviews planned to assess participants over the long term (over 12 months: Gross 2015a; Hayden 2005; Hurkmans 2009; Regnaux 2015; Saragiotto 2016; van der Heijden 2015). With chronic conditions, it would be advisable to include longer follow‐up periods (beyond 12 months post‐randomisation) as long‐term solutions may be more relevant to their control or pain management. It is also possible that initial adaptation and potential benefits as a result of an exercise intervention may take longer to manifest in comparison to a 'healthy' person due to the possible limitations in exercise intensity and progression (a training threshold) beyond which any additional physical training may be detrimental to the underlying pathophysiological mechanisms (Daenen 2015) or simply be additional physical stress with no additional physical benefit (Benton 2011).

We grouped outcome measurement points in this overview into short term (less than six months), intermediate term (six to 12 months), and long term (longer than 12 months). The broad time window for 'short term' outcomes (less than six months) is a potential source of heterogeneity as the early period is the one where time of measurement is most likely to result in variable outcomes. These initial problems could be overcome by use of standard reporting periods in exercise intervention studies (suggested four‐weekly within the 'short term' period to assess both neural adaptation and other physiological changes). This would allow review authors to use the data gathered closest to the time point they are assessing, for more accurate analyses. Additionally, by extending the follow‐up period beyond one year (long‐term follow‐up), heterogeneity may be reduced further.

Reviews generally did not enforce a minimum exercise requirement for inclusion in their review. Additionally, not all exercise sessions were supervised or baseline fitness/physical ability was assessed subjectively, and consequently it was not reported whether the intervention was fulfilled as described, or whether the dose was enough to elicit a physiological response. Studies often rely on the self‐report of participants as to the actual physical activity and exercise being undertaken, which can lead to a greater risk of bias, and reduced study quality as it is questionable as to whether the effect can be truly attributed to the intervention. This was examined in a previous review, where it was concluded that non‐subjective physical assessment should be performed where possible (Perruchoud 2014), though these still have challenges regarding implementation.

In summary, the quality of the evidence was low (third tier): within this overview we found no tier one or tier two evidence. This is largely due to the small sample sizes and potentially underpowered studies. A number of studies within the reviews had adequately long interventions, but planned follow‐up was limited to less than one year (12 months) in all but six reviews.

Interpretation of the available data, and conclusions drawn by the review authors, were appropriate, although the conclusions were sometimes stronger than warranted by the available data. Occasionally results were not discussed with regards to the quality of the evidence or risk of bias: it is important to discuss the findings in the context of the quality of the evidence, with complete transparency, as this may affect future research, and implications for patients, funders, and policy makers.

### Potential biases in the overview process

While we have attempted to include all relevant reviews in the overview process, we do concede that by only searching the Cochrane Library, and including only current Cochrane Reviews we may have missed some key literature. However previous publications have referred to the higher quality grading (high AMSTAR score) in Cochrane Reviews due to the basic criteria necessary for publication at any stage (protocol or full review) suggesting they may be the most reliable source of evidence (O'Connell 2013).

### Agreements and disagreements with other studies or reviews

This is a summary overview of current Cochrane Reviews, we are not aware of any overviews or reviews summarising non‐Cochrane reviews.

## Authors' conclusions

There is limited evidence of improvement in pain severity as a result of exercise. There is some evidence of improved physical function and a variable effect on both psychological function and quality of life. However, results are inconsistent and the evidence is low quality (tier three). Promisingly however, none of the physical and activity interventions assessed appeared to cause harm to the participants.

**Implications for practice**

**For clinicians and people with chronic pain**

The evidence in this overview suggests that the broad spectrum of physical activity and exercise interventions assessed here (aerobic, strength, flexibility, range of motion, and core or balance training programmes, as well as yoga, Pilates, and tai chi) are potentially beneficial, though the evidence for benefit is low quality and inconsistent. The most commonly reported adverse events were increased soreness or muscle pain, which subsided after several weeks of the intervention.

Physical activity and exercise may improve pain severity as well as physical function and quality of life.

**For policy makers**

The evidence showed variable results, though in some reviews there was a clinical and statistical benefit in pain relief and physical function (based on low quality evidence). The evidence suggests that physical activity or exercise is an acceptable intervention in people with chronic pain, with minimal negative adverse effects. However based on this low quality evidence, we cannot provide direction to the content of an exercise programme should clinicians decide to implement one.

**Implications for research**

There is a clear need for further research into exercise and physical activity for chronic pain in adults.

**General implications**

- Future research should report baseline values for outcome measures in both intervention and control groups, together with detailed relevant information about the participants. Knowing the baseline value is relevant to interpreting any change observed as a result of the intervention, and understanding the broader value of the intervention.
- Where possible, pain results should be reported as the number of people achieving 50%, 30%, and 10% pain relief, and the number who did not meet that point (dichotomous outcome). These are clinically important cut‐offs in pain intervention research, and reporting in this way allows readers to observe the clinical effect more effectively.
- Reporting should include median and range as well as mean and standard deviation (SD) of results. This will allow readers to review the effects of any outliers that may have skewed the data, which often goes unnoticed in the reporting of mean and SD alone.
- The importance of clear intervention reporting is underestimated: often studies report both intervention and control programmes simply, where other researchers and clinicians alike are unable to replicate the trial or intervention. Recommendations for reporting are based on the Consolidated Standards of Reporting Trials (CONSORT) statement (www.consort‐statement.org/), but this alone does not detail the extent of necessary intervention and control programmes reporting. The template for intervention description and replication (TIDieR) approach (Hoffman 2014) is intended as an extension to CONSORT item 5 ("The interventions for each group with sufficient details to allow replication, including how and when they were actually administered") and is a checklist for detailing the programmes using: why (rationale), what (materials and procedures), who, how, where, when, and how much.

**Design**

- One previous review highlighted the increased bias often present in questionnaires and other self‐report measures of physical activity in people with chronic pain, and as a result made the recommendation to use objective measures instead, such as accelerometers, or the use of direct and indirect calorimetry, where possible (Perruchoud 2014), though these still have challenges regarding implementation. This would allow direct and exact comparison and analyses of actual energy expenditure and treatment effect.

**Population/participants/sample**

- There needs to be a focus on participants with generalised and/or widespread chronic pain, instead of (or as well as) condition‐specific populations.
- Studies should include people with higher pain severity (greater than 50/100 on a 100‐point visual analogue scale) at baseline. People with mild‐moderate pain should still be included, but it would be advisable to separate the results for analysis, ensuring the study is adequately powered to allow this subgroup analysis in advance. This way we could determine if exercise has benefit overall, or affects one group more than another, and tailor exercise programmes according to the individual needs.
- It has been previously suggested that for 20% to 25% of participants undertaking an exercise programme there is little to no favourable response (Timmons 2014), while a small percentage (5% to 10%) have adverse events (Bouchard 2012). It is therefore vitally important that much larger sample sizes are used: ideally *more than 200 participants per arm*, though even this number in total would increase the quality of the evidence in the first instance. In this way we may be able to learn to identify individuals who will benefit, and those who will require further intervention.

**Interventions**

- Different forms of exercise should be researched in detail. For the purposes of this overview, we combined all physical activity and exercise interventions under one banner to determine if there was any effect. However a number of reviews separately analysed resistance (strength) training, aerobic (endurance), and combination programmes. It is important to continue to examine different modalities, but currently there is not enough high quality evidence to exclude or prioritise one specific mode (resistance, endurance, stability) or medium (land/water based), or the proportion of a combination programme to be assigned to each, as all may have individual benefits for people with chronic pain.
- Intensity of exercise, duration of individual sessions, and frequency should be investigated. It is this dose alongside duration (of the entire intervention) and adherence that may determine the actual efficacy.
- More reviews and trials should attempt to minimise intervention heterogeneity by implementing minimum and maximum requirements. Only this way will the research community be able to determine more accurately the direction and magnitude of effect of a specific programme or intervention. Many of these important restrictions can be implemented as subgroup analyses, though if this is the case it is important to have adequate study numbers (ideally 200 participants per arm or subgroup).
- Due to the chronicity and long‐term nature of the condition, physiological and psychological changes may take longer to manifest. It is widely accepted that there is a delay in muscular hypertrophy as a result of exercise, and initial gains within the first few weeks of any training programme will be as a result of neural factors (Enoka 1997); this is also in line with the grading of evidence (tier two evidence or higher requires a minimum of a four‐week intervention). This suggests that longer interventions may be necessary (eight weeks for tier one evidence), though assessing participants at regular intervals, including at four weeks, would be beneficial to examine the effect of the neural adaptation alone.

**Measurement (end‐points)**

- Randomised controlled trials with long‐term follow‐up are needed. Chronic pain is defined by its chronic nature, and therefore long‐term follow‐up of results is equally important as the initial short‐term effect (if not more so): outcomes should be assessed beyond one year after randomisation. In turn this will inform the direct effect of the intervention, as well as the proportion of the population who maintains the programme of exercise employed in the intervention, or something else under the guise of physical activity as a result of participation.
- The broad time window for 'short term' outcomes (less than six months) is a potential source of heterogeneity as the early period is the one where time of measurement is most likely to result in variable outcomes. These initial problems could be overcome by use of standard reporting periods in exercise intervention studies (suggested four‐weekly assessment within the 'short term' period to assess both neural adaptation and other physiological changes). This would allow review authors to use the data recorded closest to the time point they are assessing, for more accurate and comparable analyses.
- Outcome measures used by researchers should be standardised across trials and studies. Recommendations for selecting the most appropriate and important outcome measures to those who live with chronic pain have previously been published (Initiatives on Methods, Measurement, and Pain Assessment in Clinical Trials (IMMPACT) Consensus Recommendations, Dworkin 2005; Turk 2003).

**Other**

- It would be of interest in future research to determine the reasons for non‐participation in regular physical activity or non‐compliance to a prescribed exercise intervention in people with chronic pain, and how to overcome these barriers.
- Future Cochrane Reviews could include: exercise for chronic pain or chronic widespread pain (and not specific conditions such as osteoarthritis, fibromyalgia, etc.), and exercise for neuropathic pain. These areas have not been covered by Cochrane with an exercise or physical activity intervention.

## What's new

**Table:** 

**Date**	**Event**	**Description**
18 February 2020	Amended	Clarification added to Declarations of interest.

## History

Protocol first published: Issue 8, 2014 Review first published: Issue 1, 2017

**Table:** 

**Date**	**Event**	**Description**
9 November 2018	Amended	"Next stage expected" date extended to 2022; we assess all overviews for updating five years after publication.
18 April 2017	New citation required but conclusions have not changed	Conclusions not changed; retrospective open access.
10 April 2017	Amended	See Published notes.

## Notes

This overview review was re‐published in April 2017 with retrospective open access.
